# Lactate dehydrogenase B regulates macrophage metabolism in the tumor microenvironment

**DOI:** 10.7150/thno.58380

**Published:** 2021-06-04

**Authors:** Ann-Christin Frank, Rebecca Raue, Dominik C. Fuhrmann, Evelyn Sirait-Fischer, Carsten Reuse, Andreas Weigert, Dieter Lütjohann, Karsten Hiller, Shahzad Nawaz Syed, Bernhard Brüne

**Affiliations:** 1 Institute of Biochemistry I, Faculty of Medicine, Goethe-University Frankfurt, 60590 Frankfurt, Germany.; 2 Department for Bioinformatics and Biochemistry, BRICS, Technische Universität Braunschweig, Rebenring 56, 38106 Braunschweig, Germany.; 3 Institute of Clinical Chemistry and Clinical Pharmacology, University Hospital Bonn, 53127 Bonn, Germany.; 4 Helmholtz Centre for Infection Research, Inhoffenstraße 7, 38124 Braunschweig, Germany.; 5 German Cancer Consortium (DKTK), Partner Site Frankfurt, 60590 Frankfurt, Germany.; 6 Frankfurt Cancer Institute, Goethe-University Frankfurt, 60596 Frankfurt, Germany.; 7 Fraunhofer Institute for Translational Medicine and Pharmacology ITMP, 60596 Frankfurt, Germany.

**Keywords:** Breast cancer, LDHB, RNA therapeutics, metabolism, tumor-associated macrophages.

## Abstract

**Background**: Glucose metabolism in the tumor-microenvironment is a fundamental hallmark for tumor growth and intervention therein remains an attractive option for anti-tumor therapy. Whether tumor-derived factors such as microRNAs (miRs) regulate glucose metabolism in stromal cells, especially in tumor-associated macrophages (TAMs), to hijack them for trophic support, remains elusive.

**Methods**: Ago-RIP-Seq identified macrophage lactate dehydrogenase B (LDHB) as a target of tumor-derived miR-375 in both 2D/3D cocultures and in murine TAMs from a xenograft mouse model. The prognostic value was analyzed by ISH and multiplex IHC of breast cancer patient tissues. Functional consequences of the miR-375-LDHB axis in TAMs were investigated upon mimic/antagomir treatment by live metabolic flux assays, GC/MS, qPCR, Western blot, lentiviral knockdown and FACS. The therapeutic potential of a combinatorial miR-375-decoy/simvastatin treatment was validated by live cell imaging.

**Results**: Macrophage LDHB decreased in murine and human breast carcinoma. LDHB downregulation increase aerobic glycolysis and lactagenesis in TAMs in response to tumor-derived miR-375. Lactagenesis reduced fatty acid synthesis but activated SREBP2, which enhanced cholesterol biosynthesis in macrophages. LDHB downregulation skewed TAMs to function as a lactate and sterol/oxysterol source for the proliferation of tumor cells. Restoring of LDHB expression potentiated inhibitory effects of simvastatin on tumor cell proliferation.

**Conclusion**: Our findings identified a crucial role of LDHB in macrophages and established tumor-derived miR-375 as a novel regulator of macrophage metabolism in breast cancer, which might pave the way for strategies of combinatorial cancer cell/stroma cell interventions.

## Introduction

Aerobic glucose metabolisms, besides glutamine consumption, are of major importance to harness energy for tumor growth 1, 2. Despite steep nutrient and oxygen gradients in solid tumors, cancer cells favor aerobic glycolysis that causes lactate production, despite being less efficient in terms of ATP synthesis. It is plausible to assume that cancer cells may exploit stromal cells for their metabolic need. It has been shown that cancer-associated fibroblast serves as a lactate donor in the tumor microenvironment (TME), once they are activated by their direct contact with tumor cells 3. Whether other stroma cells such as tumor-associated macrophages (TAM), which constitute up to 50% of the total tumor mass 4,5, also functioning as lactate donors in the TME is unknown. Whereas, tumor-derived lactate has been shown to be taken by macrophages, thereby adding to a pro-tumoral phenotype shift 6, it is not known if and how tumor cells hijack the glucose metabolizing machinery of TAMs to reprogram them as lactate producers.

TAMs are a dynamic and heterogeneous immune cell population, which is associated with immune-suppressive and trophic functions that support tumor progression, invasion, and metastasis 7, 8. Thus, a high number of infiltrating TAMs often correlates with poor disease outcome 9. In response to tumor-microenvironmental cues that comprises products of an altered tumor cell metabolism, TAMs adapt their metabolism, which is closely linked to their pro- (M1-like) or anti-inflammatory (M2-like) polarization. Understanding the intricate relationship between the TME and TAMs that governs these metabolic changes represents an essential step to progress towards a TAM-directed anti-tumor therapy.

One way of tumor cell-macrophage (MΦ) cross-talk is via microRNAs (miR), which are small non-coding RNAs that attenuate target gene expression at the post-transcriptional level 10. Recently, we showed that breast tumor cells release miR-375 upon apoptosis, which are taken up by MΦ, thereby stimulating their migration and infiltration 11. Here we provide evidence that tumor cell-derived miR-375 downregulates LDHB in macrophages, which is critical for their metabolic adaptation to become tumor supportive. Lactate dehydrogenase (LDH) is one of the key enzymes in glycolysis that catalyses the bidirectional conversion of pyruvate and lactate 12, 13. The tetrameric enzyme is composed of different subunits, LDHA and LDHB. LDHA has higher affinity for pyruvate and preferentially converts pyruvate to lactate, and is overexpressed in many malignant tumors, including breast cancer 14. In contrast, there is very limited information on LDHB, which transforms lactate to pyruvate in malignant cells 15 and TAMs. We discovered a non-redundant role of LDHB in macrophages and explored its pathophysiological relevance in breast cancer. We also provide evidence for the clinical relevance of the miR-375-LDHB axis in a pre-clinical combinatorial anti-tumor treatment model.

## Methods

### Reagents

Actinomycin D, lactate and simvastatin were purchased from Sigma-Aldrich (München, Germany). All reagents were dissolved according to the manufacturer's instructions.

### Cell Culture

All cell lines were obtained from ATCC-LGC Standards GmbH (Wesel, Germany) and routinely tested for mycoplasma contamination. MCF-7, T47D and MDA-MB-231 cells were cultivated in RPMI 1640 containing 1% sodium pyruvate and 1% non-essential amino acids, 10% FCS, 100 U/mL penicillin, and 100 μg/mL streptomycin. Human MΦ were cultured in RPMI 1640 containing 5% AB-positive human serum (DRK Blutspendedienst Baden-Würtemberg-Hessen, Frankfurt, Germany), 100 U/mL penicillin, and 100 μg/mL streptomycin (MΦ media). If not stated otherwise, all cell culture supplements came from PAA Laboratories (Cölbe, Germany).

### Generation of Human MΦ from Buffy Coats and Coculture with Tumor Cells

Primary human monocytes (peripheral blood mononuclear cells, PBMCs) were isolated from commercially obtained buffy coats from anonymous healthy donors (DRK-Blutspendedienst Baden-Württemberg-Hessen, Institut für Transfusionsmedizin und Immunhämatologie, Frankfurt, Germany) using Ficoll-Hypaque (PAA Laboratories) density centrifugation as previously described 11. For coculture experiments, MΦ were cultured at a density of 5 × 10^5^ cells/mL. Tumor cells were detached from culture flasks using trypsin-EDTA, washed with PBS and resuspended in MΦ-media. Tumor cells were cocultured with MΦ at the same density. After times indicated, residual MCF-7, T47D or MDA-MB-231 cells were removed from plates using trypsin-EDTA for 3-5 min, which left adherence of MΦ unaltered.

### MiR Mimic, Antagomir and siRNA Transfection

MiR mimic, antagomir, and siRNA transfections were performed using HiPerfect (Qiagen, Hilden, Germany) according to the manufacturer's instructions. For overexpression of miR-375, primary human MΦ in six-well plates were transfected with MISSION^®^ hsa-miR-375 mimic or MISSION^®^ miR negative control 2 from *C. elegans* (cel-miR-39a; both from Sigma-Aldrich). To inhibit miR-375, primary human MΦ or tumor cell lines were transfected with MISSION^®^ has-miR-375 inhibitor (antagomir) or negative control 2 from *C. elegans* (cel-miR-243-3p; both from Sigma-Aldrich). Lactate secretion from MΦ was blocked by transfection of cells with ON-TARGETplus MCT4 siRNA or control siRNA (both from Dharmacon, Lafayette, Colorado, USA).

### Ago Immunoprecipitation, Library Generation and AGO-RIP-Seq

To identify new targets of miR-375 in MΦ, 10 × 10^6^ - 20 × 10^6^ cells were transfected with synthetic miR-375 mimic (mimic) or with nonspecific cel-miR-39a (scramble) for 48 h. Ago immunoprecipitation (Ago-IP), library generation and AGO-RIP-Seq were performed as previously described 11, 16, 17.

### GSEA Analysis

Differentially expressed genes between control and miR-375 containing macrophages (ACM treated, mimic transfected) were used as an input to analyze gene sets in the Molecular Signatures Database using GSEA 4.0.2 via the Gene Pattern Platform.

### RNA Isolation, Reverse Transcription and Quantitative Real-time PCR

RNA isolation, reverse transcription and quantitative real-time PCR were performed as previously described 11. Primers for hsa-miR-375 (MIRAP00360) and SNORD44 (MIRCP00005) were from Sigma-Aldrich. Primers for cel-miR-39a (MS00019789) and *KI67* (249900) were purchased from Qiagen. All other primers were obtained from Biomers (Ulm, Germany) and sequences are presented in **table S1**. Relative mRNA/miR expression was calculated using the CFX-Manager^TM^ v3.2 software (Bio-Rad Laboratories) and the ΔΔCt method and normalized to the respective control RNAs indicated in figure legends.

### Generation of Stable MCF-7 miR-375 Decoy and Control Cell Line

MCF-7 control and miR-375 decoy cells were generated as previously described 11. In the MCF-7 miR-375 decoy cells, the endogenous mature miR-375 is inhibited by the stable transfection of a lentiviral vector encoding for the miR-375 decoy insert (Plasmid #46617; Addgene, Cambridge, USA), while control cells were transfected with an empty vector. Transduction efficiency was analyzed based on green fluorescent protein-expression, detected by flow cytometry using a LSRII/Fortessa flow cytometer (BD Bioscience, Heidelberg, Germany).

### Generation of MCF-7 Tumor Spheroids and Coculture with Primary Human Monocytes

3D tumor spheroids from MCF-7 cells were generated by using the liquid-overlay technique as described 18. For this, 5 × 10^3^ cells per well were seeded onto non-adherent 1% agarose-coated 96-well plates and allowed to form spheroids for 4 days. Primary human monocytes were isolated from human blood PBMCs by using CD14 MicroBeads (130-050-201; positive selection) and the AutoMACS Separator system (both from Miltenyi Biotec, Gladbach, Germany). 1 × 10^5^ monocytes were added per spheroid and cocultures were maintained for 3 days to allow monocyte infiltration and differentiation to MΦ.

### Xenograft Transplantation Experiment

Mouse care and experiments involving mice were approved by and followed the guidelines of the Hessian animal care and use committee (approval number FU/1152). Xenograft transplantation was performed with 8 -12 week-old female *NMRI*-*Foxn1^nu^*mice and MCF-7 control or MCF-7 miR-375 decoy cells as previously described 11. Tumors were collected for fluorescence activated cell sorting and immunohistochemistry.

### Flow Cytometry and Cell Sorting

Single-cell suspensions were stained with fluorochrome conjugated antibodies and analyzed on a LSRII/Fortessa flow cytometer or sorted using a FACSAria III cell sorter (both from BD Biosciences) as previously described 11.

For cell sorting of MCF-7 tumors from xenograft transplantation experiments, single-cell suspensions were created using the Human Tumor Dissociation Kit and the GentleMACS Dissociator (Miltenyi Biotec). Non-specific binding was blocked with 2% of human and murine Fc receptor block (eBioscience, Frankfurt, Germany) in PBS for 15 min on ice. Cells were stained with an antibody mixture of CD11b-eFluor605 (BioLegend, San Diego, USA; #101257; 1:200 dilution), F4/80-Pe-Cy7 (BioLegend, #123114; 1:200 dilution), Ly-6G-APC-Cy7 (BioLegend, #127624; 1:100 dilution), CD326-PE (BioLegend, #324205; 1:100 dilution), CD11c-BV711 (BD Bioscience, #363048; 1:200 dilution), Ly-6c-PerCP-PE-Cy5.5 (BD Bioscience, #560525; 1:200 dilution), CD45-VioBlue (Miltenyi Biotec, #130102430; 1:50 dilution), and HLA-DR-APC (Miltenyi Biotec, #130102139; 1:50 dilution). Cell suspensions were filtered through 30 μm cell strainers and diluted to ideal concentrations for cell sorting.

### Immunoblotting

Immunoblotting of MΦ protein lysates was performed as previously described 11. The following antibodies were used at 4°C overnight: LDHB (Abcam, Cambridge, UK; #ab85319; 1:1000 dilution), LDHA (Abcam #ab101562; 1:1000 dilution), and actin (Santa Cruz Biotechnology, Heidelberg, Germany; #sc-8031; 1;3000 dilution) according to the manufacturer's instructions. Proteins were visualized by IRDye secondary antibodies using the Li-Cor Odyssey imaging system (all from LICOR Bioscience, Bad Homburg, Germany).

### *In Situ* Hybridization and Multiplex Immunohistochemistry

Tumors from xenograft mouse experiments were fixed in 4% paraformaldehyde and paraffin-embedded. 4 µm thick sections were stained using the Opal staining system, imaged by the Vectra3 automated imaging software and analyzed with inForm2.0 software using the phenotyping tool according to the manufacturer's instructions (PerkinElmer, Rodgau, Germany). Tumor sections were stained with the following antibodies: LDHB (Santa Cruz, sc-100775; 1:300 dilution); CD163 (Abcam, ab182422; 1:250 dilution); spectral 4′,6-diamidino-2-phenylindole (DAPI; PerkinElmer).

Tissue microarrays (TMAs) of human normal breast and invasive breast cancer were provided by the Cooperative Human Tissue Network and the Cancer Diagnosis Program, which are funded by the National Cancer Institute. Other researchers may have received exemplars from the same subjects. *In situ* hybridization of double DIG-labeled miRCURY LNA^TM^ miRNA Detection Probe hsa-miR-375 (Qiagen, #YD00610232) and scamble-miR (Qiagen, #YD00699004) has been performed according to the miRCURY LNA^®^ miRNA detection probes handbook with some modifications followed by staining with antibody against human LDHB (Santa Cruz, sc-100775; 1:300 dilution) and spectral DAPI (PerkinElmer) using the Opal staining system as previously described 11. Slides were imaged using the Vectra3 automated imaging system and images were analyzed using InForm2.0 (Perkin Elmer) and ImageJ software.

### Plasmid Construction

To generate a plasmid with miR-375-binding sites for human LDHB, psiCHECK 2^TM^-vector (Promega, Madison, USA) was digested using NotI and XhoI restriction enzymes (New England Biolabs, Frankfurt, Germany). The 3′-UTR of LDHB was amplified from human cDNA with primers sense 5′-TAGGCGATCGCTCGAGCTAGTGAGCTCTAGGCTG-3′ and antisense 5′-TTGCGGCCAGCGGCCGCCACACTACAATAGTTAATTTTAT-3′ (both from Biomers), and inserted into the linearized psiCHECK™-2 vector with the In-Fusion® HD Cloning Kit (Takara) according to the manufacturer's protocol.

### Luciferase Reporter Assay

For luciferase activity assay, human MΦ were transiently cotransfected with 2 μg LDHB 3′-UTR reporter plasmid or an empty control plasmid with or without MISSION® hsa-miR-375 Mimic (Sigma-Aldrich) using ViromerRED transfection reagent (Lipocalyx, Halle, Germany) as previously described 11. The activity in miR-375 cotransfected cells was expressed as fold change compared to the cells transfected with vectors only.

### Measurement of Cellular Cholesterol, Non-cholesterol Sterols, and Oxysterols

In a first set of experiments the amount of non-cholesterol sterols and oxysterols was analyzed by gas chromatography- mass spectrometry- selected ion monitoring (GC-MS-SIM). For this, 1.5 - 2 × 10^6^ MΦ were washed once with ice-cold PBS, scraped and centrifuged at 1000×g for 5 min at 4°C. The supernatants were discarded, and macrophage cell pellets were spun in a SpeedVac^TM^ concentrator (12 mbar; Savant AES 1000). Cholesterol, non-cholesterol sterols, and oxysterols were extracted using chloroform, followed by alkaline hydrolysis and the measurement of cholesterol precursor and oxysterol concentrations with GC-mass spectrometry-selected ion monitoring 19. The trimethylsilylethers of the sterols were separated on a DB-XLB (30 m length × 0.25 mm internal diameter, 0.25 μm film) column (Agilent Technologies, Waldbronn, Germany) using the 6890N Network GC system (Agilent Technologies). Non-cholesterol sterols were measured on a 5973 Network MSD (Agilent Technologies) and Epicoprostanol (Steraloids, Newport, RI, USA) and deuterium labelled oxysterols were used as an internal standard for quantification. Total cholesterol was measured by GC-flame ionization detection on an HP 6890 GC system (Hewlett Packard, Waldbronn, Germany), equipped with a DB-XLB (30 m length × 0.25 mm internal diameter, 0.25 μm film) column (Agilent Technologies) using 5α-cholestane (Steraloids) as internal standard 20.

In a second set of experiments, total cholesterol was analyzed using the Amplex Red Cholesterol Assay Kit (Life Technologies, Carlsbad, USA) according to the manufacturer's recommendations. Fluorescence was measured in a TECAN SPARK^®^ multimode microplate reader (TECAN, Männedorf, Switzerland) using excitation of 545 nm and emission of 590 nm. Protein concentrations were measured using the DC Protein Assay (Bio-Rad Laboratories) and cholesterol concentrations were reported per mg protein.

### Extraction of Metabolites and Fatty Acids for Gas Chromatography-Mass Spectrometry (GC/MS)

To measure intra- and extracellular metabolites and fatty acid methyl esters (FAMEs), 1.5 - 2 × 10^6^ cells were harvested as previously described 21. Briefly, cells were washed with sterile filtered 0.9% NaCl and quenched with 0.2 mL of -20°C methanol. An equal volume of ice-cold water containing 1 µg/mL glutaric acid (internal standard for metabolite measurement) was added and cells were collected with a cell scraper and transferred to tubes containing 0.2 mL -20°C chloroform containing 25 µM palmitate (internal standard for fatty acid measurement). Cell extracts were shaken at 1400 rpm for 20 min at 4°C (Thermomixer Eppendorf) followed by centrifugation at 16000×g for 5 min at 4°C. 0.2 mL of upper aqueous phase and 0.15 mL of lower non-polar phase were collected in specific glass vials with micro inserts.

For extraction of extracellular metabolites, 15 µL of cell supernatant was transferred to tubes containing 135 µL of a mixture of 1:9 methanol (-20°C)/Millipore water with 1 µg/mL glutaric acid as internal standard. Extracts were shaken at 2000 rpm for 10 min at 4°C and centrifuged at 17000×g for 10 min at 4°C. 60 µL of supernatant was transferred to glass vials with micro inserts. Extracted metabolites and fatty acids were evaporated under vacuum at -4°C using a CentriVap Concentrator (Labconco Corporation, Kansas City, USA).

### Metabolite Measurement

Metabolite derivatization was performed using a Gerstel MPS. Dried polar metabolites were dissolved in 15 µL of 2% methoxyamine hydrochloride in pyridine and shaken for 60 min at 40°C. An equal volume of N-tert-butyldimethylslyl-N-methyltrifluoroacetamide (MTBSTFA) was added and held for 60 min at 40°C. 1 µL of sample was injected into an SSL injector at 270°C in splitless mode. GC/MS analysis was performed using an Agilent 7890A GC equipped with a 30-m DB-35MS 5-m Duraguard capillary column. As carrier gas helium was used at a flow rate of 1.0 mL/min. The GC oven temperature was held at 100 °C for 2 min and increased to 300 °C at 10 °C/min. After 3 min, the temperature was increased to 325 °C. The GC was connected to an Agilent 5975C inert XL MSD, operating under electron ionization at 70 eV. The MS source was held at 230 °C and the quadrupole at 150 °C. The MS was operated in selected ion monitoring. The total run time of one sample was 25.00 min. All GC/MS chromatograms were processed by using Metabolite Detector software 22. Protein concentrations were determined using the DC Protein Assay (Bio-Rad Laboratories) and data were normalized to protein content of the respective sample.

### FAME Measurement

Dried non-polar fatty acids were dissolved in 500 μl 2% (w/v) H_2_SO_4_ in methanol and incubated at 50°C for 90 min. Afterwards, 100 μl saturated NaCl solution and 200 μl hexane were added, followed by vigorously mixing and short centrifugation. The last steps were repeated three times and the upper phase was transferred to a GC-MS vial. The hexane was evaporated under vacuum and samples were re-dissolved in 50 μL hexane and vials were caped immediately. 1 μL of sample was injected into an SSL injector at 270 °C in splitless mode. GC/MS analysis was performed using an Agilent 7890A GC equipped with a 30-m DB-35MS 5-m Duraguard capillary column. Helium was used as carrier gas at a flow rate of 1.0 mL/min. The GC oven temperature was held at 55°C for 5 min and increased to 325 °C at 6 °C/min. The GC was connected to an Agilent 5975C inert XL MSD, operating under electron ionization at 70 eV. The MS source was held at 230 °C and the quadrupole at 150 °C. The MS was operated in scanning monitoring. The total run time of one sample was 60 min. All GC/MS chromatograms were processed by using Metabolite Detector software. Protein concentrations were determined using the DC Protein Assay (Bio-Rad Laboratories) and data were normalized to protein content of the respective sample.

### Live Metabolic Flux Assays

The extracellular acidification rate (ECAR) and the cellular oxygen consumption rate (OCR) were analyzed using a Seahorse 96 extracellular flux analyzer (Agilent). 20,000 MCF-7 control or decoy cells or 30,000 human MΦ were seeded per well of a Seahorse 96-well cell culture plate at the day of measurement and equilibrated in Krebs Henseleit buffer (111 mM NaCl, 4.7 mM KCL, 1.25 mM CaCl_2_, 2 mM MgSO_4_, 1.2 mM Na_2_HPO_4_) supplemented with 3 mM L-glutamine 0.5 h before measurement. For ECAR measurement, Glycolysis Stress Test was performed: Cells were treated with 5 mM glucose (Sigma-Aldrich), 2.5 µM oligomycin (Oligo; Cayman Chemical) to block ATP-coupled respiration, and 50 µM 2-deoxyglucose (2-DG; Cayman Chemical) to inhibit glycolysis through competitive binding to glucose hexokinase. For OCR measurement, Mito Stress Test was performed: Cells were treated with 2.5 µM oligomycin, 1 µM carbonylcyanide m-chlorophenylhydrazone (CCCP; Sigma-Aldrich) to uncouple the respiratory chain, and 1 µg/mL antimycin A (AA; Sigma-Aldrich) together with 1 µM rotenone (Rot; Cayman Chemicals) to block mitochondrial respiration.

### Lactate Assay

To measure intra- and extracellular lactate amounts the lactate assay from Sigma-Aldrich (MAK064) was used. Briefly, 0.5 - 1×10^6^ cells were homogenized in 200 µL Lactate Assay Buffer and centrifuged at 13,000×g for 10 min. 200 µL of cell supernatants were centrifuged at 1,000×g for 5 min to remove cells followed by another centrifugation at 2,000×g for 10 min to remove cells debris. Both, cell samples as well as cell media were deproteinized with a 10 kDa MWCO spin filter (Merck) to remove lactate dehydrogenase. Afterwards, the lactate assay was performed according to the manufacturer's guidelines. Protein concentrations were determined using the DC Protein Assay (Bio-Rad Laboratories) and lactate concentration was reported per mg protein.

### Cell Proliferation Assays

In a first set of experiments, the Incucyte^®^ S3 Live cell Analysis System (Essen Bioscience) and the Incucyte software were used to measure MCF-7 cell proliferation upon coculture with MΦ. Pictures of cocultures were taken every 6 h for a total of 162 h and cell number was determined based on the GFP signal intensity.

In a second set of experiments, fluorescence signal of MCF-7 cells was measured in a TECAN SPARK^®^ multimode microplate reader (TECAN, Männedorf, Switzerland) every 24 h for a total of 144 h and cell proliferation was determined based on the GFP signal intensity.

### Statistical Analysis

All data are presented as mean values ± SEM of at least three independent experiments. Before normalization, all data were pre-analyzed to determine normal distribution and equal variance with D'Agostino-Pearson omnibus normality test. Parametric versus non-parametric tests were applied accordingly, as indicated in the figure legends. Statistical analyses of non-normalized data were performed using two tailed Student's *t*-test, and or two-way analysis of variance with Bonferroni's correction. Normalized data was analyzed using one sample *t* test or Wilcoxon rank-sum test. Asterisks indicate significant differences between experimental groups (*p < 0.05; **p < 0.01; **p < 0.001).

## Results

### Regulation of LDHB in Primary Human MΦ by Tumor Cell-Derived MiR-375

**Figure 1:**
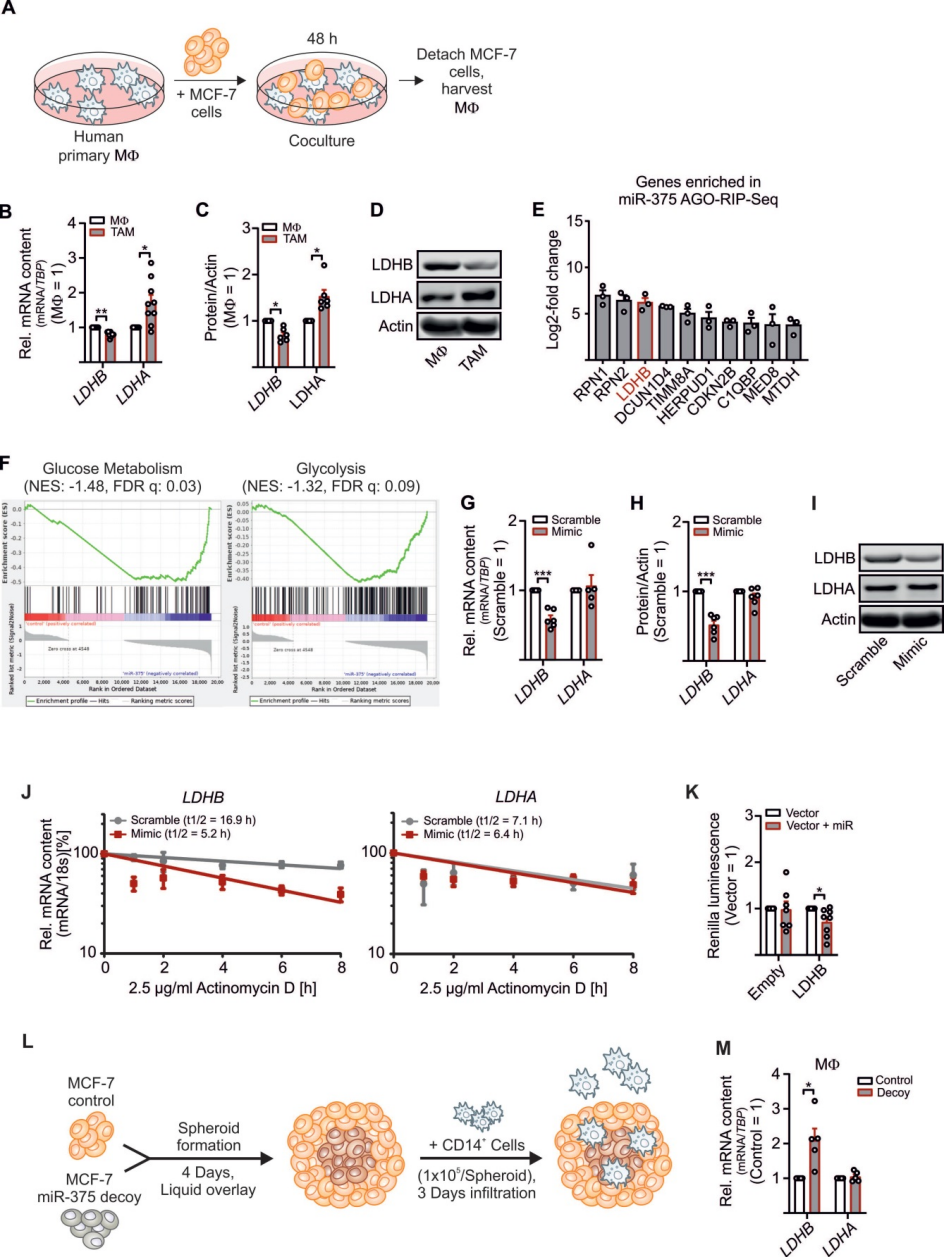
** Regulation of *LDHB* by tumor-derived miR-375 in human MΦ.** Primary human MΦ were cocultured with MCF-7 cells for 48 h. (**A**) Experimental scheme. (**B**) mRNA expression of *LDHB* and *LDHA* in TAMs relative to control MΦ. (**C**) Protein expression of LDHB and LDHA in TAMs relative to control MΦ. Actin was used for normalization. (**D**) Representative Western blots of LDHB, LDHA and actin. (**E, F**) Primary human MΦ were transfected with synthetic miR-375 mimic or cel-miR-39a (scramble) for 48 h, followed by AGO-IP and RNA-Sequencing. (**E**) Genes enriched in miR-375 containing MΦ in AGO-RIP-Seq. (**F**) Representative gene set enrichment plots of gene sets enriched in miR-375 containing MΦ. (**G** - **J**) MΦ were transfected with synthetic miR-375 mimic or cel-miR-39a (scramble) for 48 h. (**G**) *LDHB* and *LDHA* mRNA expression and (**H**) protein expression of LDHB and LDHA relative to scramble transfected MΦ. Actin was used for normalization. (**I**) Representative Western blots of LDHB, LDHA and actin. (**J**) MΦ were treated with actinomycin D. *LDHB* and *LDHA* contents at 0 h treatment were set to 100%. mRNA half-life (t_1/2_) was calculated by exponential regression curve. (**K**) MΦ were transfected with *LDHB* 3'UTR reporter plasmid or empty control vector with or without synthetic miR-375 mimic for 48 h. Binding of miR-375 to LDHB was analyzed as the ratio of Renilla luciferase to firefly luciferase activity. (**L**, **M**) Coculture of MCF-7 tumor spheroids with human CD14^+^ monocytes for 3 days. (**L**) Experimental design. (**M**) Cocultures were harvested and MΦ were separated from MCF-7 cells via anti-CD14 microbeads. mRNA expression of *LDHB* and *LDHA* relative to MΦ from MCF-7 control spheroids is shown. Data are represented as mean ± SEM of *n* ≥ 3. *p*-values were calculated using Wilcoxon rank-sum test (**B**, **C**, **K**, **M**) and one-sample *t* test (**G, H**). *, *p* < 0.05; ***, *p* < 0.001.

To explore how tumor-derived factors affect TAM metabolism, we set up a coculture of primary human MΦ with MCF-7 breast cancer cells. Upon their interactions, MΦ get polarized towards a TAM phenotype (**Figure 1A**). This allowed to investigate alternations in key glycolytic enzymes involved in lactate production in the TME. As expected, LDHA mRNA expression (**Figure 1B**) and protein amount (**Figure 1C-D** and **S1A**) increased, while the mRNA expression of LDHB significantly decreased in the TAM fraction upon 48 h of coculture (**Figure 1B-D** and **S1A-C**). LDHB is known as a direct target of miR-375 in certain cancer cells 23, 24. We provided evidence that apoptotic MCF-7 cells release miR-375, which is taken up by MΦ via CD36 and accumulating intracellularly (**Figure S1D**) 11. Whether miR-375 targets *LDHB* in stroma cells, particularly in MΦ, is unknown. To answer this question, we investigated the tumor-derived miR-375 targetome in primary human MΦ using AGO-RIP-Seq upon transfection with miR-375 mimic and immunoprecipitation with a pan-Ago antibody 11, 16. *LDHB* was elevated in the AGO-RIP-Seq dataset (**Figure 1E**). Gene set enrichment analysis (GSEA) suggested that genes involved in glycolysis and glucose metabolism are overrepresented in miR-375 containing MΦ (**Figure 1F**). Overexpression of miR-375 with a synthetic miR-mimic (**Figure S1E**) decreased LDHB mRNA (**Figure 1G**) and protein expression (**Figure 1H-I,** and **Figure S1A**), while LDHA mRNA and protein amounts remained unaltered. MiRs regulate gene expression by degradation of target mRNAs and/or through translational inhibition 25. Therefore, MΦ overexpressing miR-375 were treated with actinomycin-D to block transcription, which destabilized *LDHB* mRNA, while *LDHA* mRNA stability was unaffected. Suggestively miR-375 decreases *LDHB* mRNA stability in MΦ (**Figure 1J**). To verify these results MΦ were transfected with reporter vectors containing the 3'UTR sequence of *LDHB* behind a Renilla Luciferase coding region. Luciferase activity was reduced when MΦ were co-transfected with miR-375 mimic (**Figure 1K**). To confirm these results in a more pathophysiological setting, we generated tumor spheroids from MCF-7 control and MCF-7 miR-375 decoy cell lines 11 (**Figure S1F**) followed by infiltration with CD14^+^ human peripheral blood monocytes (**Figure 1L**). After 3 days, *LDHB* mRNA expression appeared higher in MΦ from MCF-7 decoy spheroid cocultures (**Figure 1M**), which correlated with reduced miR-375 transfer (**Figure S1F**). These results establish regulation of *LDHB* in human MΦ by tumor-derived miR-375.

### LDHB regulates Aerobic Glycolysis and Lactate Production in MΦ and MCF-7 Cells

**Figure 2:**
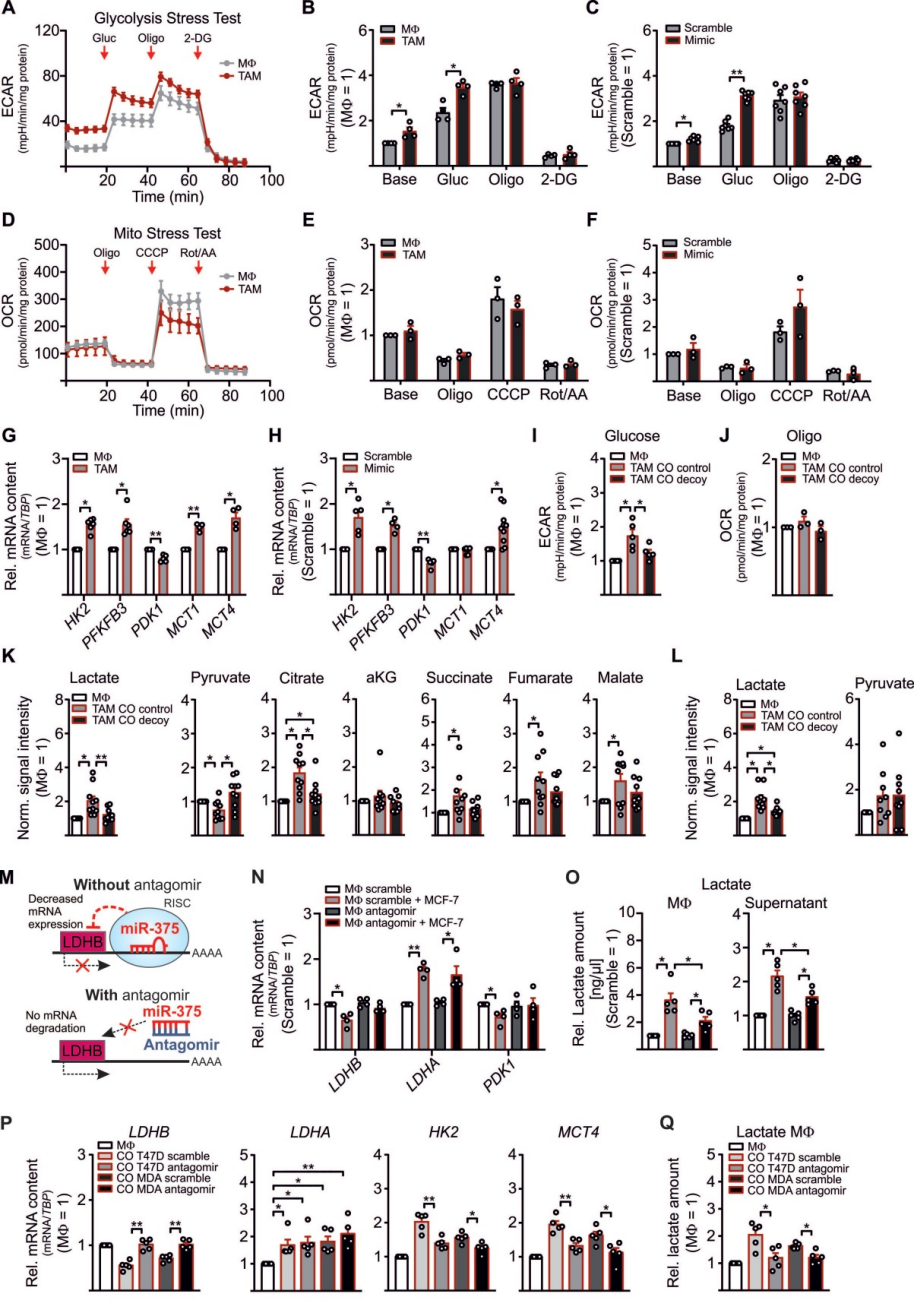
**LDHB downregulation enhances glycolysis and lactate production in human MΦ**. (**A - H**) MΦ were cocultured with MCF-7 cells (**A, B, D, E, G**) or treated with miR-375 mimic or cel-miR-39a (scramble) (**C, F, H**) for 48 h. Extracellular acidification rate (ECAR) and oxygen consumption rate (OCR) were measured by Glycolysis Stress Test and Mito Stress Test, respectively. (**A**) Representative line graphs of the mean ± SEM of ECAR. Cells were treated with 5 mM glucose (Gluc), 2.5 µM oligomycin (Oligo), and 50 µM 2-deoxyglucose (2-DG). (**B**) ECAR of TAMs relative to control MΦ. (**C**) ECAR of miR-375 mimic transfected MΦ relative to scramble transfection. (**D**) Representative line graphs of the mean ± SEM of OCR. Cells were treated with 2.5 µM Oligomycin (Oligo), 1 µM carbonyl cyanide m-chlorophenylhydrazone (CCCP) and 1 µg/mL antimycin A (AA) together with 1 µM rotenone (Rot). (**E**) OCR of TAMs relative to control MΦ. (**F**) OCR of miR-375 mimic transfected MΦ relative to scramble transfection. (**G, H**) mRNA expression of *HK2*, *PFKFB3*, *PDK1*, and *MCT1*/*MCT4* in TAMs (**G**) and mimic transfected MΦ (**H**), relative to respective controls. (**I, J**) MΦ were cocultured with MCF-7 control or decoy cells for 48 h and ECAR upon glucose treatment (**I**), and OCR upon oligomycin treatment (**J**) was measured in TAMs. Data are relative to control MΦ. (**K, L**) MΦ were cocultured with MCF-7 control or MCF-7 decoy cells for 48 h. Intracellular (**K**) and extracellular (**L**) metabolites were extracted from MΦ and measured by GC/MS. Data are normalized to control MΦ. (**M - O**) MΦ were transfected with synthetic miR-375 antagomir or cel-miR-243-3p (scramble) for 24 h followed by coculture with MCF-7 cells for another 48 h. (**M**) Experimental scheme. (**N**) mRNA expression of *LDHB*, *LDHA* and *PDK1* relative to scramble transfected MΦ. (**O**) MΦ intra- and extracellular lactate amount was measured by a lactate assay kit and normalized to scramble transfection. (**P**, **Q**) T47D or MDA-MB-231 cells were transfected with scramble or miR-375 antagomir for 24 h followed by coculture with MΦ. (**P**) mRNA expression of *LDHB*, *LDHA*, *HK2*, and *MCT4* as well as lactate amount (**Q**) in TAMs was measured. Data are represented as mean ± SEM of *n* ≥ 3 and *p*-values were calculated using Wilcoxon rank-sum test *, *p* < 0.05; **, *p* < 0.01; ***,* p* < 0.001.

Although the role of LDHB in macrophages is underappreciated, in pancreatic cancer a low LDHB expression induces glycolysis 26. There is also evidence for a robust shift from oxidative phosphorylation to glycolysis in murine and human TAMs 27-30. To investigate whether downregulation of LDHB by miR-375 in MΦ might induce glycolysis, we performed metabolic flux assays with TAMs and miR-375 overexpressing MΦ. The extracellular acidification rate (ECAR) was enhanced in MCF-7 cocultured TAMs (**Figure 2A-B**) and mimic transfected MΦ (**Figure 2C**, **Figure S2A**), while the oxygen consumption rate (OCR) remained unaltered (**Figure 2D-F, Figure S2B**). Expression of key glycolytic enzymes, i.e. hexokinase 2 (*HK2*), 6-phosphofructo-2-kinase/fructose-2,6-biphosphatase (*PFKFB3*), and the monocarboxylate transporters 1 and 4 (*MCT1*, *MCT4*) increased in TAMs (**Figure 2G**) and mimic transfected MΦ (**Figure 2H**) (except for *MCT1*, which was unaffected in mimic transfected MΦ). As expected, the established miR-375 target pyruvate-dehydrogenase kinase 1 (*PDK1*) was downregulated 31. We then verified these results in MΦ cocultured with control or decoy MCF-7 cells. An enhanced ECAR was corelated with enhanced mature miR-375, however, pre-miR-375 expression was unaffected in cocultured TAMs (**Figure S2C**), while decoy MCF-7 cells provoke no increase in TAM glycolysis (**Figure 2I**). OCR remained unaltered in TAMs from both cocultures (**Figure 2J**).

Based on metabolic flux assays data with MΦ, we expected similar findings in MCF-7 cells owing to their constitutive miR-375 expression and LDHB suppression. Indeed, ECAR was reduced in MCF-7 decoy cells at the basal level and upon glucose treatment (**Figure S2D**), which was in line with increased *LDHB* but reduced *PFKFBR3* mRNA expression (**Figure S2E**). However, transcript expression of *LDHA*, *MCT1*, and *MCT4* remained unaltered. Furthermore, *PDK1* mRNA increased in decoy cells, pointing towards a direct role of miR-375 in *PDK1* downregulation. These findings provide evidence for a miR-375-LDHB axis to enhance aerobic glycolysis in breast cancer cells and to provoke a glycolytic shift in TAMs.

Often, an increase in aerobic glycolysis results in excessive lactate formation 32. Since we observed enhanced glycolysis in TAMs and increased expression of the lactate transporter *MCT4*, which is induced by high lactate amounts and oxidative stress 33, 34, we asked whether miR-375 induces lactate formation in TAMs. Therefore, MΦ were cocultured with control and decoy MCF-7 cells followed by qPCR and GC-MS based metabolome analysis. In contrast to increased *LDHA*, the mRNA expression of *LDHB* and *PDK1* decreased in TAMs from control as compared to TAMs from decoy cocultures (**Figure S2F**). Correspondingly, there was a 2-fold increase of intracellular lactate in TAMs from control cocultures as compared to naïve MΦ (naïve MΦ lactate revealed a mean signal intensity of 0.07), while MCF-7 decoy cells did not stimulate lactate formation (**Figure 2K**). In addition, we observed reduced pyruvate but enhanced citrate, succinate, fumarate, and malate levels in TAMs from control cocultures (**Figure 2K**). TAMs share metabolic features with LPS/IFN‐γ‐activated M1-like MΦ, where an enhanced glycolytic flux along with an increased flux through PDH due to reduced PDK1 activity provokes citrate and succinate accumulation 35-39. Besides reduced serine amounts in TAMs, we did not detect alterations in other selected intracellular metabolites (**Figure S2G**). Moreover, extracellular lactate significantly increased in control cocultures (in naïve MΦ lactate revealed a mean signal intensity of 3.43) (**Figure 2L**), while extracellular pyruvate remained the same. Interestingly, valine and serine levels were reduced in coculture supernatants (**Figure S2H**). Since lactate was low in the supernatants from decoy cocultures, we asked whether this might be due to diminished lactate release from MCF-7 decoy cells. Intracellular lactate was reduced by roughly 30% in MCF-7 decoy as compared to control cells (**Figure S2I**), while pyruvate increased. Nevertheless, the amount of extracellular lactate and pyruvate remained the same in control and miR-375 decoy cancer cells (**Figure S2J**), suggesting that miR-375-induced LDHB downregulation indeed fosters lactate production in TAMs.

To further substantiate these findings, MΦ were transfected with miR-375 antagomir prior to coculture. We hypothesized that in TAMs the presence of the antagomir should prevent tumor-derived miR-375 binding to *LDHB*, thereby enhancing *LDHB* expression and reducing lactate formation (**Figure 2M**). As to our expectation, antagomir treatment rescued downregulation of *LDHB* and *PDK1* mRNA in TAMs, while *LDHA* mRNA expression was still evident (**Figure 2N**). Accordingly, intra- and extracellular lactate amounts were reduced in antagomir-transfected TAMs (**Figure 2O**). To rule out that these results are cell line specific, we cocultured MΦ with scramble or miR-375 antagomir transfected T47D or MDA-MB-231 breast cancer cells. In tumor cells, antagomir treatment significantly reduced the miR-375 amount in both cell lines (**Figure S2K**), which was in line with increased *LDHB* and reduced *PFKFB3* mRNA expression, while LDHA remained unaltered (**Figure S2L**). Moreover, antagomir transfected tumor cells showed reduced intracellular lactate levels as compared to scramble transfected cells, while extracellular lactate remained constant upon antagomir transfection (**Figure S2M**). Accordingly, in tumor cell antagomir treatment decreased miR-375 amounts in TAMs upon coculture and lowered LDHB expression, enhanced glycolytic activity as indicated by the enhanced mRNA expression of HK2 and MCT4 (**Figure 2P**), as well as enhanced lactate synthesis (**Figure 2Q**). These findings suggest that tumor cells use miR-375 to hijack MΦ, thereby increasing lactate production and secretion.

### LDHB Downregulation Drives MΦ Polarization and Subsequent Tumor Growth

**Figure 3:**
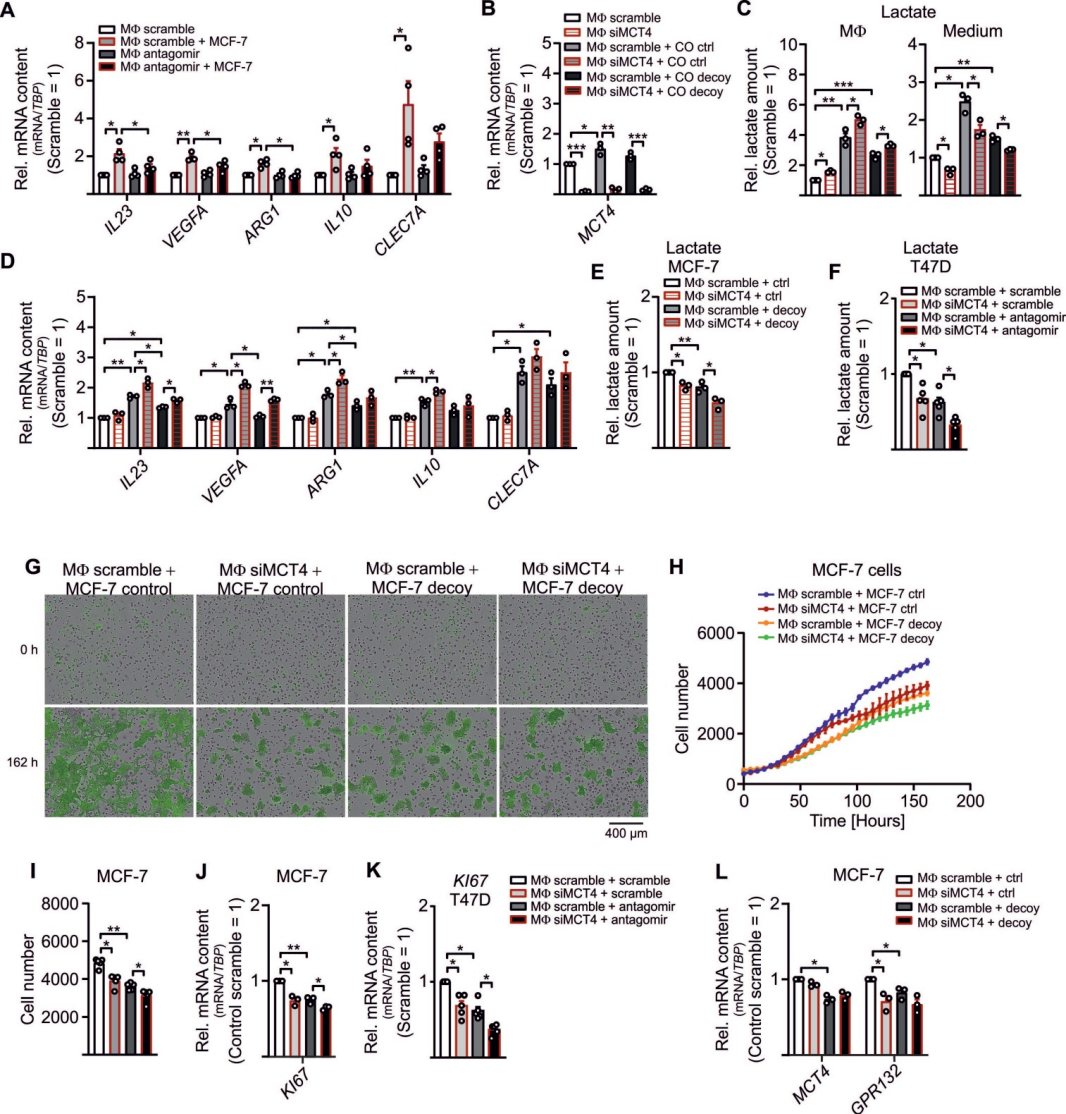
** Lactate drives MΦ polarization and enhances tumor cell proliferation**. (**A**) MΦ were transfected with miR-375 antagomir or cel-miR-243-3p (scramble) for 24 h followed by coculture with MCF-7 cells for another 48 h. Quantitation of *IL23*, *VEGFA*, *ARG1*, *IL10*, and *CLEC7A* relative to scramble transfected MΦ is shown. (**B - J**) MΦ were transfected with non-specific siRNA or siRNA against *MCT4* for 24 h followed by coculture with MCF-7 cells (**B - E, K**) or T47D cells (**F, L**) for 48 h or 168 h (**F - H**). (**B**) *MCT4* expression relative to scramble transfected MΦ is shown. (**C**) Lactate amount in MΦ and in the media was measured by lactate assay kit and normalized to scramble transfected MΦ. (**D**) Quantitation of genes involved in MΦ polarization relative to scramble transfected MΦ is shown. (**E, F**) MCF-7 cells (**E**) or T47D cells (**F**) were harvested from cocultures and intracellular lactate amount was measured by a lactate assay kit. Data are normalized to MCF-7 control/T47D scramble cells from cocultures with scramble transfected MΦ. (**G - I**) MCF-7 cell proliferation upon MΦ coculture was analyzed by IncuCyte live cell imaging system. (**G**) Representative pictures. (**H, I**) The number of MCF-7 cells was determined. (**J - L**) Quantitation of proliferation marker *KI67* (**J, L**) as well as *MCT4*, and *GPR132* (**K**) relative to control MCF-7 cells from cocultures with scramble transfected MΦ is shown. Data are represented as mean ± SEM of *n* ≥ 3 and *p*-values were calculated Wilcoxon rank-sum test *, *p* < 0.05; **, *p* < 0.01; ***,* p* < 0.001.

With this set of experiments, we aimed to explore functional roles of increased lactate formation due to LDHB downregulation in TAMs. Tumor cell-derived lactate promotes MΦ polarization by inducing vascular endothelial growth factor (VEGF) and arginase 1 (ARG1) expression and fosters MΦ IL-23/IL-17 production 6, 40. Corroborating these findings, treatment of MΦ with lactate increased mRNA expression of *IL23*, *VEGFA*, *ARG1*, and *MCT4*, but not *IL10* or *CLEC7A* (dectin-1) (**Figure S3A**). Upregulation of these factors i.e. *IL23*, *VEGFA*, and *ARG* in TAMs was abolished in antagomir transfected TAMs (**Figure 3A**). *IL10* and *CLEC7A,* increased in TAMs, but remained unaltered upon antagomir treatment. Apparently, miR-375 is important for the lactate-mediated phenotype switch, but not for the classical TAM polarization 11. Next, we asked whether these effects can be further enhanced when lactate is accumulating in TAMs. Knockdown of the *MCT4* lactate exporter in TAMs 41 (**Figure 3B**) increased intracellular lactate accumulation (**Figure 3C**), while extracellular lactate was reduced. In addition, lactate was apparently lower in TAMs from decoy cocultures as compared to TAMs from control cocultures, which correlated with decreased *IL23*, *VEGFA*, and *ARG* mRNA expression (**Figure 3D**). This could be rescued by a KD of MCT4 in TAMs from decoy cocultures.

Since lactate was also released into the supernatant, we asked whether MΦ-derived lactate can be taken up by tumor cells to foster proliferation 42-44. Intracellular lactate was diminished in MCF-7 as well as T47D cells isolated from MCT4 KD MΦ cocultures (**Figure 3E, F**), suggesting that MΦ-derived lactate is taken up by tumor cells. In MCF-7 decoy and T47D antagomir cells the lactate amount was low upon coculture, which was due to both, reduced lactate formation by decoy cells as well as reduced MΦ lactate secretion.

We then measured GFP^+^ tumor cell proliferation upon coculture with MΦ using the IncuCyte^TM^ live cell imaging system. Proliferation of MCF-7 decoy cells was reduced as compared to MCF-7 controls (**Figure 3G-I**) and was further suppressed when MΦ lactate secretion was attenuated. This was supported by decreased mRNA expression of the proliferation marker *KI67* (**Figure 3J**). These results were replicated in T47D cells, when T47D cells were transfected with antagomir and cocultured with MΦ in which lactate secretion was blocked, resulted in reduced *KI67* mRNA expression (**Figure 3K**. Interestingly, expression of glycolytic enzymes *HK2*, *PFKFBR3*, and *PDK1* decreased in cocultured MCF-7 cells when lactate release from MΦ was blocked (**Figure S3B**). Furthermore, mRNA expression of *GPR132,* which is the key lactate sensor in tumor cells and MΦ 45, decreased in cocultured tumor cells from MCT4 KD MΦ (**Figure 3L**), while *MCT4* mRNA expression remained unaltered. Evidently, miR-375 downregulates LDHB in MΦ, which stimulates their lactate formation and secretion to foster tumor cell proliferation.

### Downregulation of LDHB Activates SREBP2 and Cholesterol Biosynthesis in MΦ

**Figure 4:**
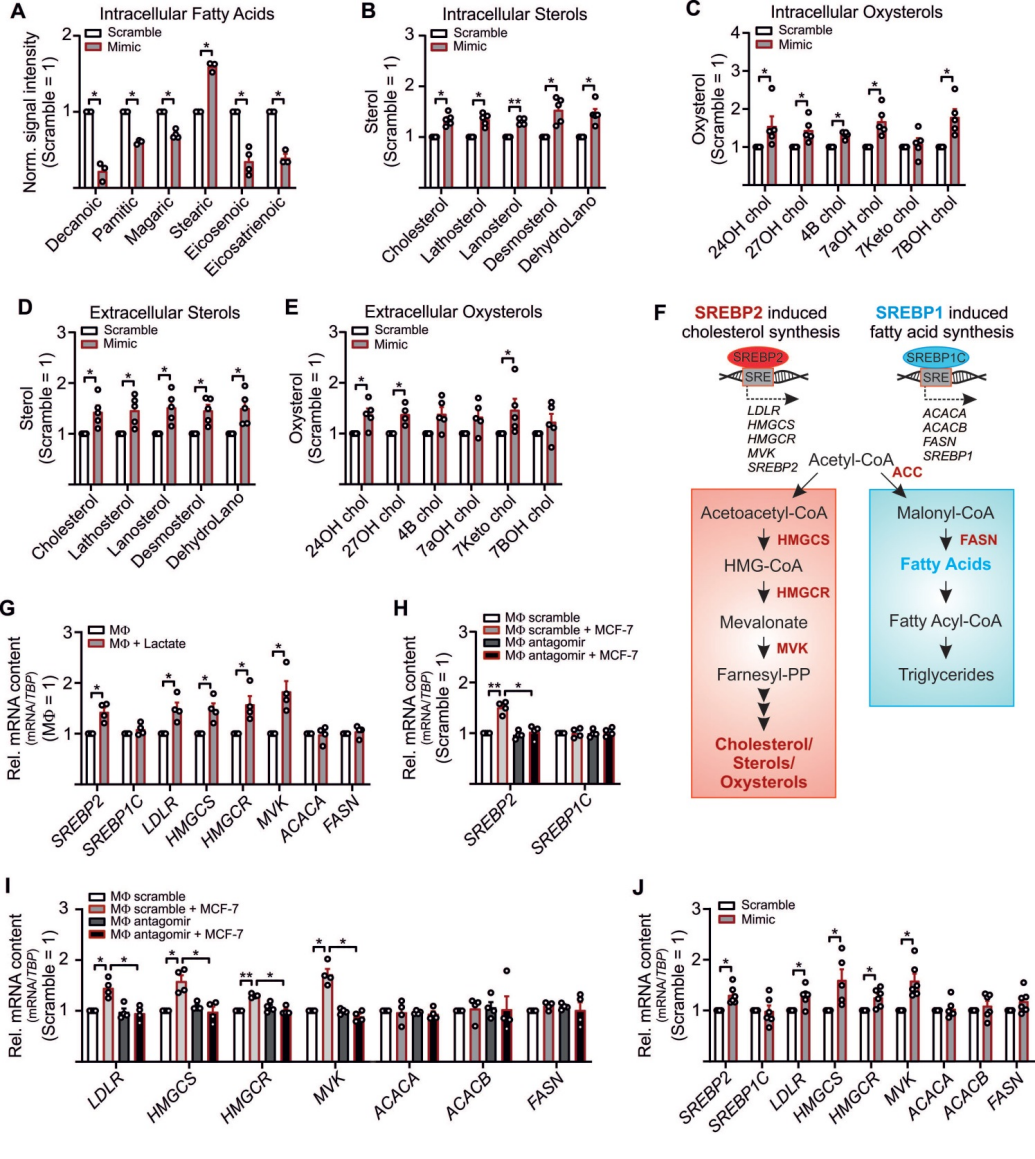
** MiR-375-mediated LDHB downregulation induces SREBP2 activation and cholesterol biosynthesis in MΦ**. (**A - E**) MΦ were treated with synthetic miR-375 mimic or cel-miR-39a (scramble) for 48 h. Data are normalized to scramble transfected MΦ. (**A - E**) Intracellular fatty acids (**A**), cholesterol, non-cholesterol sterols (**B**), and oxysterols (**C**) were measured by GC/MS. (**D, E**) Extracellular cholesterol and non-cholesterol sterols (**D**), and oxysterols (**E**) were measured by GC/MS. (**F**) Overview of *SREBP2*/*SREBP1* mediated gene regulation and cholesterol as well as fatty acid synthesis. Key enzymes are highlighted in red. (**G**) MΦ were treated with 10 mM lactate for 24 h. Quantitation of *SREBP1*/*SREBP2* mRNA and their target genes relative to untreated MΦ. (**H, I**) MΦ were transfected with synthetic miR-375 antagomir or cel-miR-243-3p (scramble) for 24 h followed by coculture with MCF-7 cells for another 48 h. mRNA expression of *SREBP1*/*SREBP2* (**H**) and their target genes (**I**) relative to scramble transfected MΦ. (**J**) mRNA expression of *SREBP2/SREBP1* and their targets. Data are represented as mean ± SEM of *n* ≥ 3 and *p*-values were calculated using Wilcoxon rank-sum test *, *p* < 0.05; **, *p* < 0.01.

Besides the notion that lactate is one factor that adds to tumor cell proliferation, other factors, such as stroma cell-derived cholesterol, might be of relevance as well 46, 47. Since we noticed an inverse correlation between lactate and pyruvate (**Figure 2L**) and pyruvate serves as an indirect precursor for free fatty acids and sterol synthesis, we expected reduced free fatty acid and sterol production in LDHB downregulated TAMs. To follow this assumption, cell pellets and supernatants from miR-375 mimic transfected MΦ were harvested to measure and fatty acids, sterols and oxysterols by GC-MS. Palmitic acid, magaric acid, eicosenoic acid, and eisosatrienoic acid decreased in MΦ overexpressing miR-375, while the level of stearic acid was enhanced (**Figure 4A**). Whereas several other fatty acids measured remained unaltered (**Figure S4A**), we noticed an increase of intracellular (**Figure 4B, C**) and extracellular (**Figure 4D, E**) sterols and oxysterols upon miR-375 overexpression.

Higher levels of cholesterol/oxysterols besides lactate accumulation were unexpected. However, it has been demonstrated that an acidic pH, based on lactate accumulation, can trigger activation and nuclear translocation of the transcription factor SREBP2. SREBPs are key regulators of lipid homeostasis 48, 49 with three isoforms existing: SREBP1a activates fatty acid- and cholesterol synthesis, SREBP1c induces fatty acid synthesis, and SREBP2 provokes cholesterol synthesis 50 (**Figure 4F**). Since miR-375 caused lactate accumulation in TAMs we speculated whether this might trigger SREBP2 activation and the concomitant expression of cholesterol biosynthetic enzymes 51, 52. Exposing MΦ to lactate indeed increases *SREBP2*, but not *SREBP1C* mRNA (**Figure 4G**). Also, mRNA expression of SREBP2 target genes low-density lipoprotein receptor (LDL), HMG CoA synthase (HMGCS), HMG Co A reductase (HMGCR), and mevalonate kinase (MVK) increased upon lactate treatment, while the SREBP1C target genes acetyl-CoA carboxylase A (ACACA) and fatty acid synthase (FASN) remained unaltered (**Figure 4G**). In TAMs, *SREBP2* (**Figure 4H**) and SREBP2 target gene expression (**Figure 4I**) were enhanced, and these responses were ablated when miR-375-induced lactate formation was blocked by antagomir treatment. *SREBP1C* expression remained unaltered. Furthermore, *SREBP2* mRNA expression was decreased in TAMs from miR-375 decoy cocultures (**Figure S4B**). The knockdown of MCT4 in TAMs did not enhance *SREBP2* expression upon coculture with control or mR-375 decoy MCF-7 cells (**Figure S4B**), suggesting that interfering with lactate export cannot further induce SREBP2 activation. Supporting evidence came from experiments when *SREBP2* and SREBP2 target gene expression was enhanced in miR-375 overexpressing MΦ (**Figure 4J**), while *SREBP1C* and its target genes as well as citrate synthase (CS), ATP citrate synthase (ACLY), and GPR132 mRNA expression remained unaltered (**Figure S4C**). To conclude, miR-375-induced lactate accumulation activates SREBP2, thereby enhancing cholesterol biosynthesis in TAMs.

### LDHB Caused Cholesterol Production, Which Fostered Tumor Cell Proliferation

**Figure 5:**
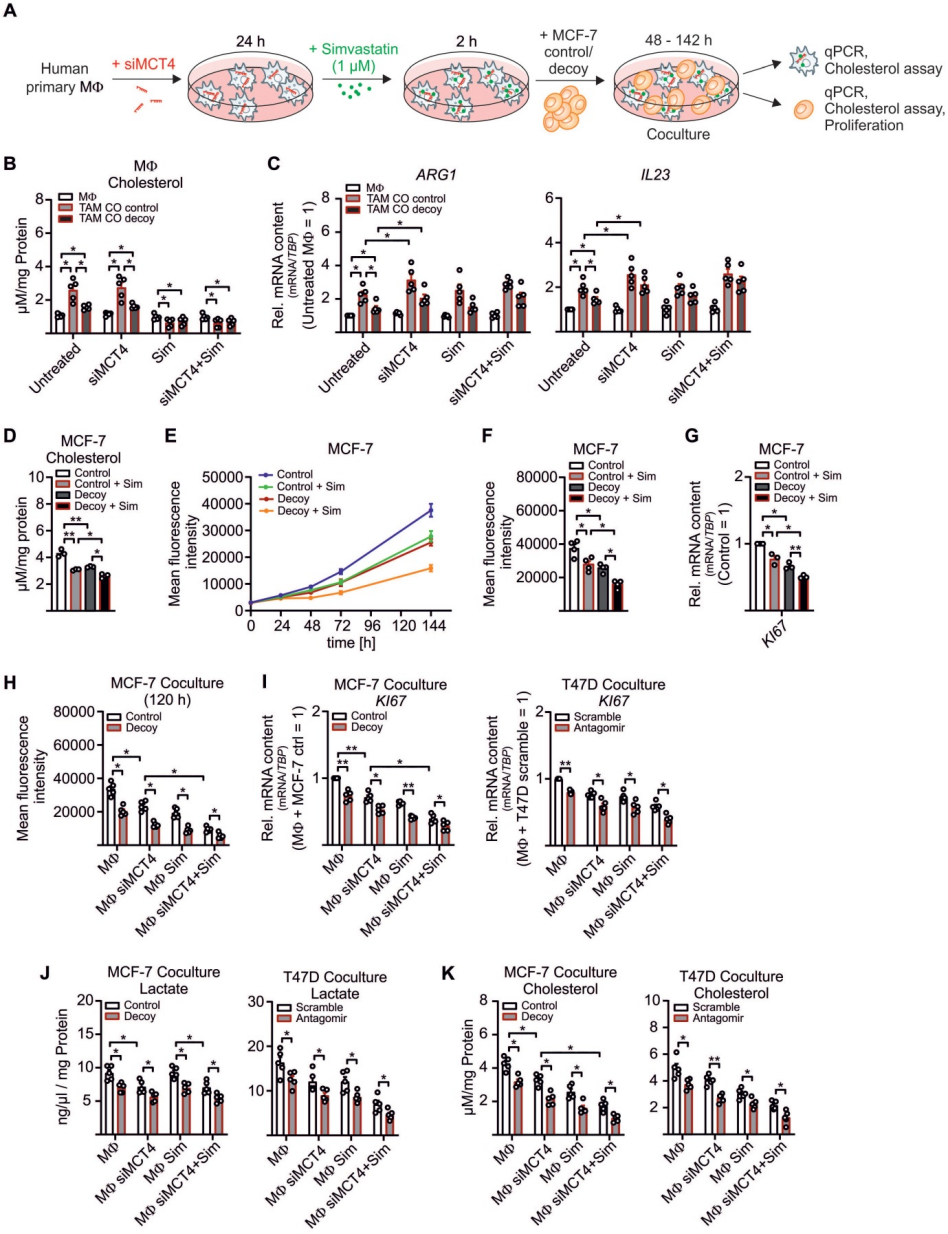
** MΦ-derived cholesterol enhances breast tumor cell proliferation.** (**A - C**) MΦ were transfected with non-specific siRNA or siRNA against *MCT4* for 24 h followed by pre-treatment with 1 µM simvastatin (Sim) or DMSO for 2 h. MΦ were washed and cocultured with MCF-7 cells for another 48 h to 144 h. (**A**) Experimental scheme. (**B**) Total cholesterol was measured in MΦ using the AmplexRed cholesterol assay kit. (**C**) mRNA expression of *ARG* and *IL23* in MΦ relative to DMSO treated scramble transfected MΦ. (**D - G**) MCF-7 control and decoy cells were treated with 1 µM simvastatin or 1 µM DMSO as control for 2 h followed by additional culture for up to 144 h. (**D**) Total cholesterol was measured in MCF-7 cells after 48 h. (**E, F**) GFP^+^ MCF-7 cell proliferation was measured using the TECAN reader. (**E**) Line graphs showing MCF-7 cell proliferation. (**F**) Mean fluorescence intensity at 144 h is shown and representative for the number of MCF-7 cells. (**G**) MCF-7 cells were harvested and mRNA expression of *KI67* relative to untreated control MCF-7 cells is shown. (**H - K**) MCF-7 and T47D cells from the experiment displayed in A were analyzed. (**H**) Proliferation of GFP^+^ MCF-7 cells from cocultures was measured based on the mean fluorescence intensity of GFP. (**I**) mRNA expression of *KI67* relative to untreated control MCF-7/scramble T47D cells. (**J**) Lactate amount in MCF-7 and T47D cells was quantified using the lactate assay kit. (**K**) Total cholesterol in MCF-7 and T47D cells was measured using the AmplexRed assay. Data are represented as mean ± SEM of *n* ≥ 3 and *p*-values were calculated using Wilcoxon rank-sum test (**B, C, G**) and two-way ANOVA with Bonferroni's correction (**D, F, H - K**). *, *p* < 0.05; **, *p* < 0.01.

We now aimed to investigate the functional relevance of enhanced cholesterol biosynthesis and/or the increased sterol/oxysterol efflux in TAMs. Before setting up the coculture, lactate export from MΦ was blocked by MCT4 KD (**Figure S5A**), and MΦ were treated with the HMGCR inhibitor simvastatin for 2 h to block cholesterol biosynthesis 53 (**Figure 5A, Figure S5B**). Tumor cell-derived miR-375 enhanced cholesterol accumulation in TAMs, whereas the increase in lactate, due to the *MCT4* KD, did not potentiate cholesterol synthesis (**Figure 5B**). The *MCT4* KD enhanced *ARG* and *IL23* mRNA expression in TAMs, while simvastatin left their mRNA expressions unaltered (**Figure 5C**). We concluded that downregulation of LDHB triggered lactate formation to stimulate TAM pro-tumor functions (**Figure 3A**, **S3A, 5C**), while enhanced cholesterol biosynthesis did not alter the TAM phenotype.

Since cholesterol accumulates in various cancers 54 and cholesterol-depleting agents reduce proliferation and apoptosis 55, 56, we hypothesized that MΦ-derived cholesterol might induce MCF-7 cell proliferation. First, we established that MCF-7 cell growth indeed correlated with the cholesterol content. Therefore, control and decoy MCF-7 cells were pre-treated with simvastatin and cultured for 48 h to 144 h. Cholesterol content was low in MCF-7 decoy as compared to control cells at 48 h and were further reduced by simvastatin (**Figure 5D**). We noticed a positive correlation between cholesterol content and proliferation at 144 h, starting from 48 h onwards (**Figure 5E-F**). These results were supported by reduced *KI67* mRNA expression in MCF-7 decoy as well as in simvastatin treated cells (**Figure 5G**), implying that cholesterol is necessary for tumor growth. Accordingly, both, a KD of *MCT4* in MΦ as well simvastatin treated MΦ reduced tumor cell proliferation (**Figure 5H**). Proliferation was further decreased when both MΦ lactate secretion and cholesterol biosynthesis were blocked. Again, this was supported by reduced *KI67* mRNA expression in both MCF-7 cells as well as T47D cells (**Figure 5I**). As already shown in before, the *MCT4* KD in MΦ reduced lactate amounts in MCF-7 control and decoy cells as well as in T47D scramble and antagomir transfected cells (**Figure 5J**). Interestingly, both the *MCT4* KD in MΦ as well as simvastatin treatment of MΦ significantly reduced the cholesterol content in MCF-7 and T47D cells (**Figure 5K**). These findings provide evidence for a positive feedback loop where miR-375 hijacks TAM metabolism to change their phenotype to become lactate and cholesterol producers to fuel tumor cell lactate requirement and growth.

### MiR-375 Decreased MΦ LDHB in Murine and Human Breast Carcinoma

**Figure 6:**
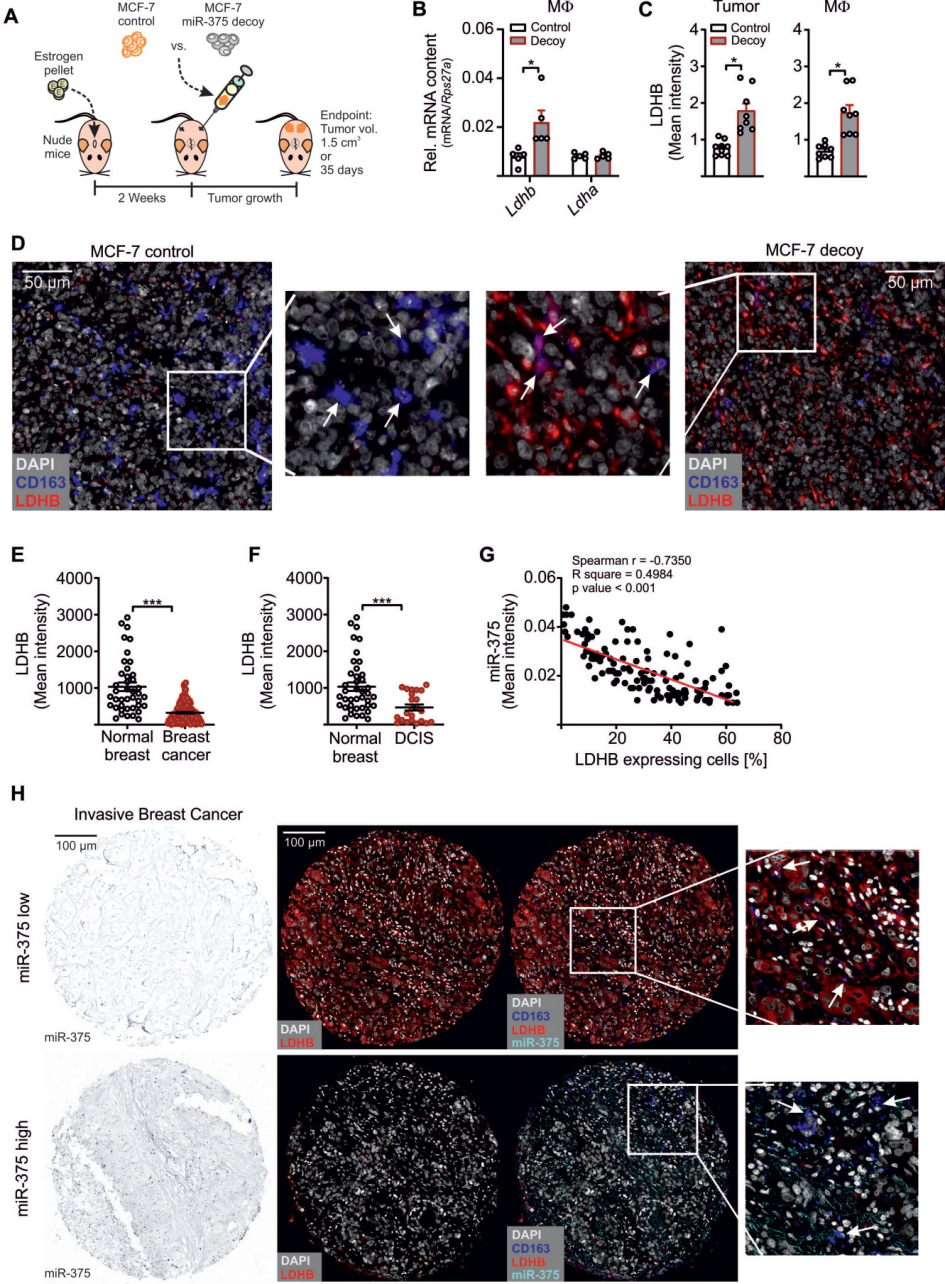
**MiR-375 decreased MΦ LDHB in mouse and human breast carcinoma**. (**A - D**) Female NMRI-*Foxn1^nu^* mice were pre-treated with 17β-estradiol pellets followed by subcutaneous injection of 1 × 10^7^ MCF-7 control or decoy cells in the right and left flank. Tumors were harvested after 35 days or after a maximum tumor volume of 1.5 cm^3^ has been reached. (**A**) Experimental layout. (**B**) Infiltrating murine MΦ were FACS-sorted out of tumors and analyzed for *Ldhb* and *Ldha* mRNA expression. (**C, D**) Immunohistochemical staining of tumor sections. (**C**) LDHB protein expression in whole tumor tissue and in infiltrating MΦ. (**D**) Representative pictures of MCF-7 control and decoy tumor sections with arrowheads in magnification showing colocalization of CD163 and LDHB. (**E - H**) Human invasive mammary carcinoma TMA sections were analyzed for miR-375 abundance by *in situ* hybridization, followed by staining of CD163 and LDHB. Bright-field signal of miR-375 was converted to fluorescence image using InForm2.0. (**E**) Mean LDHB intensity in human invasive breast cancer sections compared with normal breast (*n* = 156 breast tumors; *n* = 49 normal breasts). (**F**) Mean LDHB intensity in human ductal carcinoma *in situ* (DCIS) sections compared with normal breast (*n* = 16 DCIS; *n* = 49 normal breasts). (**G**) Correlation between miR-375 mean intensity and LDHB expressing cells in invasive breast tumor sections (n = 155). (**H**) Representative pictures of invasive breast cancer section with arrowheads in magnification showing miR-375 colocalization with CD163 and LDHB. Data are represented as mean ± SEM of *n* ≥ 3 and *p*-values were calculated using Wilcoxon rank-sum test (**B**, **C**) and two-tailed Student's *t*-test (**E, F**). *, *p* < 0.05, ***, *p* < 0.001.

In a final set of experiments, we asked whether *LDHB* is a target of miR-375 *in vivo*. Control or miR-375 decoy MCF-7 cells were injected into the flanks of female NMRI-*Foxn1^nu^* mice (**Figure 6A**). After 35 days, tumors were harvested and MΦ were FACS-sorted from tumors. In analogy to our *in vitro* findings, the miR-375 amount was low in MΦ from decoy tumors (**Figure S6A**). This correlated with higher *LDHB* expression relative to MΦ from control tumors (**Figure 6B**). Tumor sections were then stained for the MΦ marker CD163 and LDHB using the PhenOptics multispectral imaging system. LDHB protein expression increased in whole tissue sections from decoy tumors and in infiltrated TAMs (**Figure 5C**). Even though the number of infiltrating MΦ was low, we observed a colocalization of CD163 and LDHB in decoy tumors, suggesting the presence of LDHB protein in TAMs under conditions when tumor cells are low in miR-375 (**Figure 6D**).

As an early event in cancer development and progression LDHB is downregulated in different cancers, including breast cancer 15, 57-59, which is linked with unfavourable patient survival 60, 61. Since those studies unfortunately focussed on LDHB expression only in whole tumor tissues, we measured the TAM-specific miR-375 and LDHB content in tissue microarray slides of mammary carcinoma patients as previously described 11. There was a more intense staining for miR-375 in invasive breast cancer (**Figure S6B**) and ductal carcinoma *in situ* sections (**Figure S6C**) compared with normal breast tissue. Higher miR-375 staining referred to a reduced LDHB content (**Figure 6E-F**). We also observed a negative correlation between miR-375 and LDHB in tumor sections with Pearson's *r* = -0.7350 and *p* < 0.001 (**Figure 6G**). Importantly, in tumor sections showing high miR-375 amounts, LDHB was barely expressed in TAMs and *vice-versa* (**Figure 6H**). These data substantiated a link between miR-375 and LDHB in TAMs and pointed to a clinical relevance of our findings.

## Discussion

Our study adds to the molecular mechanism how breast tumor cells alter TAMs metabolism for trophic needs and homeostasis by downregulating LDHB in mouse and human breast cancer tissue. Since functional consequences of LDHB in MΦ are unknown, we expected that downregulating LDHB by miR-375 and subsequent lactagenesis may have consequences not only for TAMs but also tumor cells. Live metabolic flux assays as well as transcript analysis of glycolytic enzymes revealed that LDHB suppression via miR-375 increased aerobic glycolysis in tumor cells and TAMs, without changing OCR. This appears interesting since ECAR is a combined measure of glycolysis and the TCA cycle and lactate has been shown to be a primary source of energy for the TCA cycle 43. We observed enhanced intra- and extracellular lactate in TAMs due to miR-375 accumulation, which was accompanied, enforced *MCT4* expression. Both, MCT1 and MCT4 are upregulated in breast cancer 62. While MCT1 is required for lactate import, MCT4 is induced by high lactate and oxidative stress, to facilitate lactate secretion and to enhance the glycolytic flux 33, 34, 63. It has been shown that aerobic glycolysis enhanced by TAMs confers apoptosis resistance to breast cancer cells 64. Furthermore, *LDHB* suppression due to promoter hypermethylation has already been shown to induce a glycolytic transition in pancreatic cancer 26. LDHB is upregulated in triple-negative breast cancer cells and its knockdown reduced cell proliferation 15. Unlike LDHA, LDHB is negligibly expressed in luminal breast cancer cells like MCF-7 and its knockdown had no major effect on cell proliferation 15. In fact, MCF-7 have been reported to generate about 80% of their energy through mitochondrial respiration 65. This disparity of glucose utilization by different breast cancer types and the differential expression of LDHB makes it an attractive target for anti-tumor treatment.

We provide evidence that in addition to tumor-derived lactate, tumor cells use miR-375 to enhance lactagenesis in TAMs for tumor-promoting functions. MΦ-derive lactate enhances tumor cell proliferation (**Figure 5**), which was in line with a previous study showing that in breast cancer cells reduced intracellular lactate was due to LDHA inhibition and diminished tumor growth 66. High concentrations of lactate in biopsies of breast cancers are associated with an increased risk for developing metastasis, and a poor survival prognosis in cancer patients 67. Since lactate can be taken up even in aerobic region of tumor 67 and can be used as an alternative energy supply to fuel the TCA-cycle 68,69, our results imply that tumor cells, via miR-375-LDHB, reprogram MΦ making them lactate producers to feed tumor cell energy requirements.

We found that the accumulation of lactate activates SREBP2 but not SREBP1 in TAMs. SREBP2 then induced the expression of cholesterol biosynthetic enzymes as well as its own transcription (**Figure 6**) 48, which was possibly due to increased SREBP2 processing or alternated vesicle targeting 70, 71. While SREBP1 has already been shown to be critical for cancer progression 72, the role of SREBP2 in carcinogenesis, especially in TAMs, is unclear. Interestingly, SREBP2 is upregulated in breast and prostate cancer 73-75 and increases glycolysis 76, which is in line with enhanced glycolysis and SREBP2 expression in TAMs from our experiments. We found that enhanced sterol/oxysterol amounts in MΦ did not inhibit cholesterol biosynthesis in TAMs, which was in line with a study showing that in malignant cells the cholesterol feedback loop is non-functioning 77. In a recent study, cholesterol efflux from TAMs, accompanied by reduced intracellular cholesterol levels, induced IL-4 signalling and pro-tumoral functions 46. Accumulation of sterols and oxysterols together with their enhanced secretion did not affect the MΦ phenotype in our experiments. However, simvastatin treatment of TAMs reduced cholesterol levels in tumor cells and inhibited MCF-7 cell proliferation upon coculture, especially in miR-375 decoy tumor cells. Indeed, high levels of serum cholesterol are associated with increased breast cancer risk 78 and enhanced tumor growth and metastasis in a murine MMTV-PyMT model 79. In addition, due to their high cholesterol content, breast cancer cell lines are more sensitive to statin induced apoptosis than their healthy counterparts 56, 80. In our experiments, MΦ-derived lactate was taken up by tumor cells and added to increased tumor cell cholesterol content, suggesting that MΦ-derived lactate and cholesterol potentiated their effects to enhance tumor cell proliferation.

We measured intracellular fatty acids in TAMs with the assumption that reduced pyruvate may translate in reduced acetyl-CoA, which is an indirect precursor for fatty acid synthesis. Indeed, levels of intracellular fatty acid were low in TAMs because the level of pyruvate was reduced due to LDHB downregulation (**Figure 2K, 6F**). When pyruvate availability becomes limited, cells can use β-oxidation to produce acetyl-CoA 81, which was in line with enhanced citrate amount in TAMs from control cocultures. We observed a reciprocal regulation of lactate and pyruvate in TAMs. However, most of our mechanistic data focused on lactate but it is likely that some of the effects that we noticed might also be due to reduced levels of pyruvate. Furthermore, the preferential fate of acetyl-CoA towards fatty acid and/or sterol pathways (**Figure 4F**) is not established, especially since lactate can also be a substrate of the TCA cycle 43. These intricate relationships between pyruvate - lactate - citrate - acetyl CoA needs further investigation to better understand the role of tumor-derived miR-375 in TAMs metabolism.

Metabolic reprogramming of cells of the TME is an attractive option to improve cancer therapy 82. Despite carbohydrate-restricted diets demonstrated remarkable benefits in cancer patients 83, targeting glycolytic enzymes such as LDHA had limited success. Targeting stroma cells such as TAMs for the miR-375-LDHB axis, using RNA therapeutics, pose an interesting option since it has been demonstrated that targeting a TAM-specific long non-coding RNA inhibits glycolysis 64, and aptamers for cell surface proteins can be used to deliver siRNA into macrophages *in vivo* 84, 85. Furthermore, it has been shown that the use of statin as adjuvant breast cancer therapy reduced cancer related mortality 86. The MASTER Study (MAmmary Cancer STatin ER Positive Study) aimed at improving prognosis of ER+ breast cancer patients added statin to the current treatment regimen (ClinicalTrials.gov Identifier: NCT04601116). Similarly, there are about 43 registered clinical trials for the use of statins alone or as adjuvant therapy in breast cancer. However, there is no study that explores the role of statins with RNA therapeutics such as antagomirs. Furthermore, there are no registered trials for specifically targeting TAMs with statins despite a study clearly shows that macrophages are required for cholesterol metabolites to mediate metastasis of breast cancer 87. Our study provides a rationale to target TAMs with RNA therapeutics in combinatorial therapy with statins for breast cancer treatment.

## Supplementary Material

Supplementary figures and tables.
