# Supplementary material for: Lactate dehydrogenase B regulates macrophage metabolism in the tumor microenvironment
Source: Theranostics. 2021 Jun 4;11(15):7570–88. doi: 10.7150/thno.58380 (PMC8210612; doi:10.7150/thno.58380)
Supplement: Supplementary file 1 — Supplementary figures and tables. [file thnov11p7570s1.pdf]

SUPPLEMENTARY INFORMATION

Lactate dehydrogenase B Regulates tumor-associated macrophage metabolism in tumor microenvironment.

Ann-Christin Frank *et al.,*

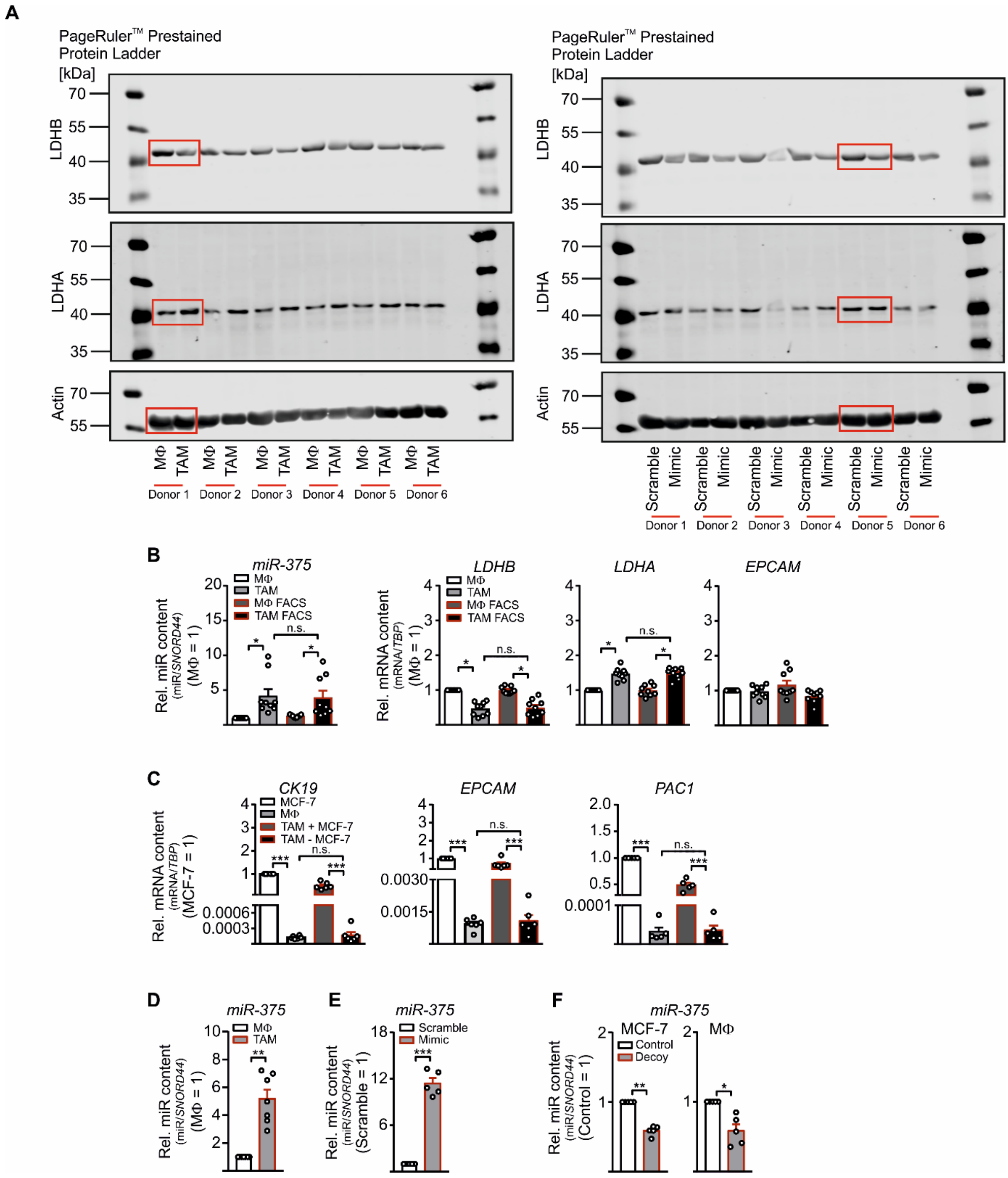

Figure S1. Regulation of *LDHB* by tumor-derived miR-375 in human MΦ, related to Figure. 1. (**A**) Uncropped blots used in Fig. 1d, i). (**B**, **C**) Validation of the purity of the TAM fractions upon coculture experiments. (**B**) MΦ were cocultured with MCF-7 breast carcinoma cells for 48 h. MCF-7 cells were removed by Trypsin/EDTA (MΦ, TAM) or whole cocultures were harvested and stained for CD326 and CD45, followed by FACS-sorting of the macrophage fraction (MΦ FACS, TAM FACS). miR/mRNA expressions of miR-375, LDHB,

LDHA, and EPCAM are shown. (**C**) MΦ were cocultured with MCF-7 cells expressing the puromycin resistance gene for puromycin N-acetyltransferase (PAC1) for 48 h. The expression of cytokeratin 19 (CK19), EPCAM and PAC1 were measured in MCF-7 cells (MCF-7), naïve control macrophages (MΦ), in whole cocultures (TAM + MCF-7) and in the TAM fraction, from which MCF-7 cells were removed (TAM – MCF-7). (**D**) Primary human MΦ were cocultured with MCF-7 cells for 48 h. miR-375 abundance in MΦ relative to control MΦ is shown. (**E**) MΦ were transfected with synthetic miR-375 mimic or cel-miR-39a (scramble) for 48 h. miR- 375 expression relative to scramble transfected MΦ is shown. (**F**) Relative miR-375 abundance in MCF-7 control and decoy spheroids and in MΦ from spheroid cocultures. Data are normalized to respective controls. Data are represented as mean ± SEM of $n \geq 3$ and p-values were calculated using Wilcoxon rank-sum test *, $p < 0.05$; **, $p < 0.01$; ***, $p < 0.001$.

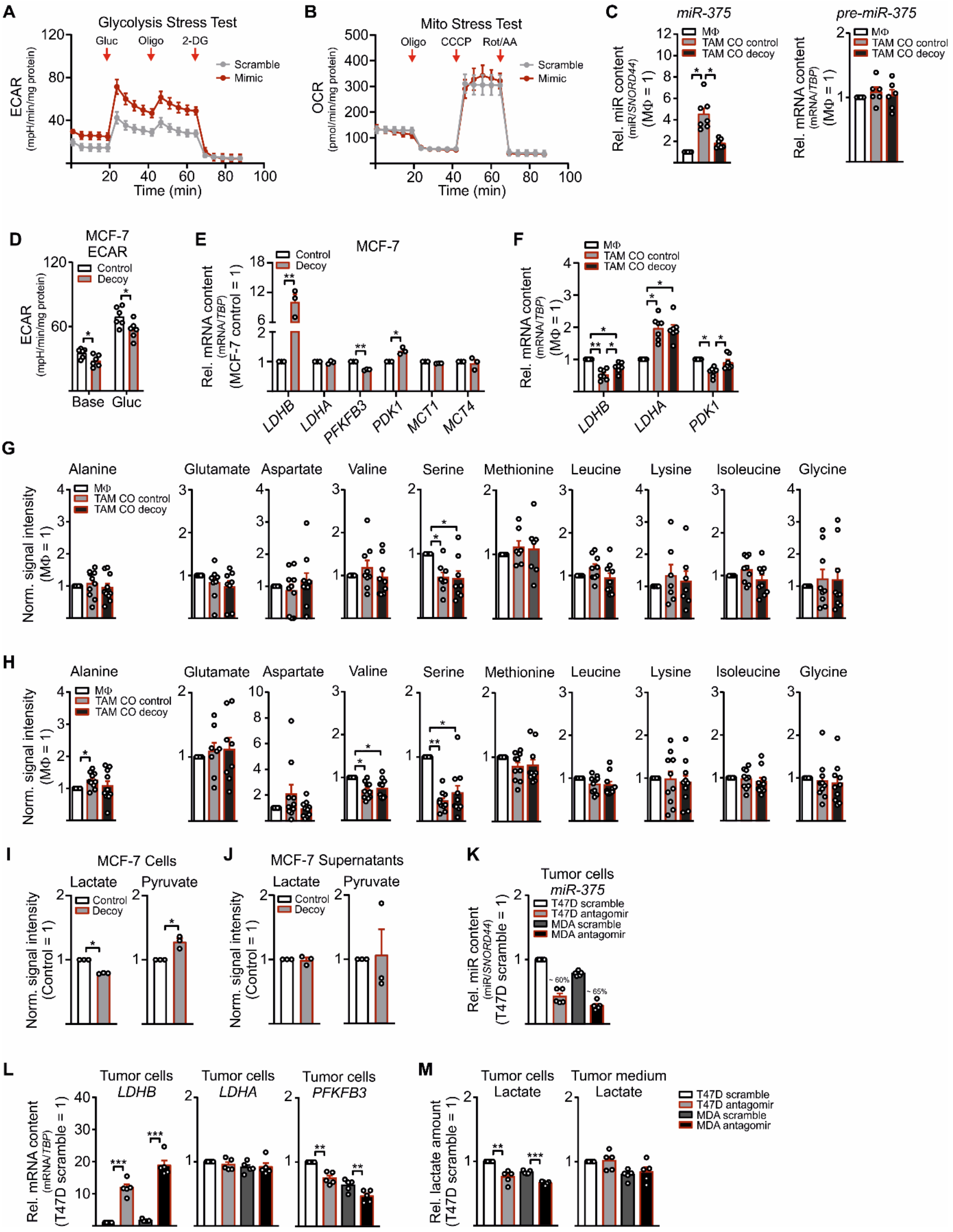

Figure S2. LDHB downregulation enhances glycolysis and lactate production in human MΦ, related to Figure 2. (**A, B**) MΦ were treated with synthetic miR-375 mimic or cel-miR-39a (scramble) for 48 h. A Glycolysis Stress Test (**A**) and Mito Stress Test (**B**) were performed. (**A**) Representative line graphs of the mean ± SEM of ECAR. Cells were treated with 5 mM

glucose (Gluc), 2.5 µM oligomycin (Oligo) and 50 µM 2-deoxyglucose (2-DG). (**B**) Representative line graphs of the mean ± SEM of OCR. Cells were treated with 2.5 µM oligomycin (Oligo), 1 µM carbonyl cyanide m-chlorophenylhydrazone (CCCP) and 1 µg/ml antimycin A (AA) together with 1 µM rotenone (Rot). (**C**) MΦ were cocultured with MCF-7 control or miR-375 decoy cells for 48 h. Mature miR-375 and pre-miR-375 abundance relative to control MΦ are shown. (**D**) ECAR of MCF-7 control and MCF-7 miR-375 decoy cells was measured. Cells were treated with 5 mM glucose (Gluc). (**E**) MCF-7 control and MCF-7 decoy cells were analyzed for the mRNA expression of *LDHB*, *LDHA*, *PFKFB3*, *PDK1*, *MCT1*, and *MCT4*. Data are normalized to expression in control MCF-7 cells. (**F**) MΦ were cocultured with MCF-7 control or miR-375 decoy cells for 48 h. *LDHB*, *LDHA*, and *PDK1* mRNA expression relative to control MΦ is shown. (**G, H**) MΦ were cocultured with MCF-7 control or MCF-7 decoy cells for 48 h. Intracellular (**G**) and extracellular (**H**) metabolites were extracted and measured by GC/MS. Signal intensities (peaks) are normalized to control MΦ. (**I**, **J**) Intracellular (**I**) and extracellular (**J**) metabolites were extracted from MCF-7 cells and measured by GC/MS. Data are normalized to control MCF-7 cells. (**K – M**) T47D and MDA-MB-231 cells were transfected with scramble or miR-375 antagomir for 24 h. miR-375 (**K**) as well as *LDHB*, *LDHA*, and *PFKFB3* (**L**) were measured by qPCR and data are normalized to T47D scramble. (**M**) Intracellular (cells) and extracellular (medium) lactate amount was measured and data are normalized to T47D scramble. Data are represented as mean ± SEM of $n \geq 3$ and p-values were calculated using Wilcoxon rank-sum test *, $p < 0.05$; **, $p < 0.01$; ***, $p < 0.001$.

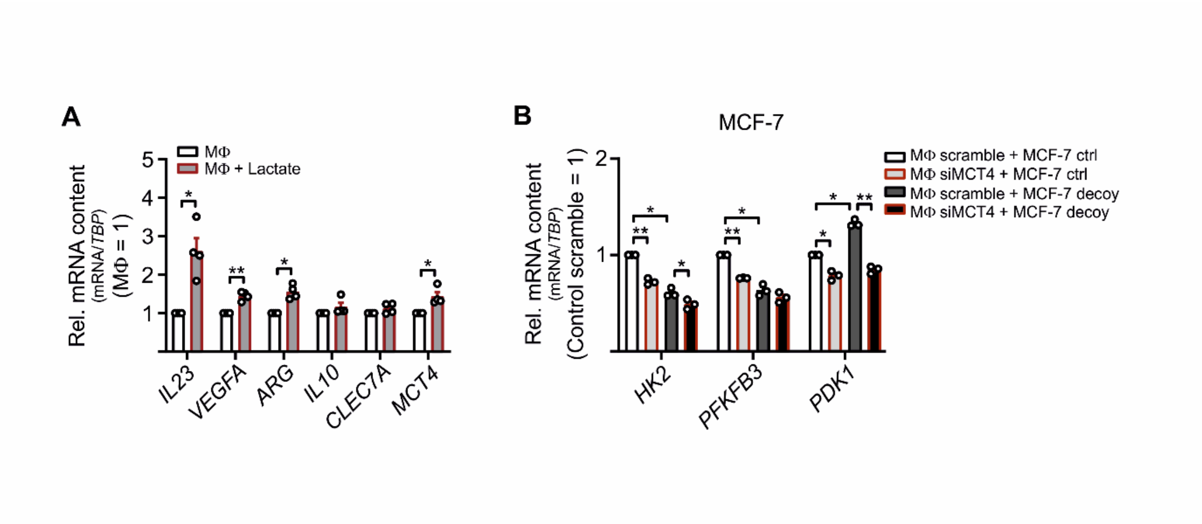

Figure S3. Lactate drives MΦ polarization and enhances tumor cell proliferation, related to Figure 3. (**A**) Primary human MΦ were treated with 10 mM lactate for 48 h and the expression of interleukin-23 (*IL23*), vascular endothelial growth factor A (*VEGFA*), arginase (*ARG*), interleukin-10 (*IL10*), and dectin-1 (*CLEC7A*), and monocarboxylate transporter 4 (*MCT4*) was measured by qPCR. Data are normalized to untreated control MΦ. (**B**) MΦ were transfected with non-specific siRNA or siRNA against MCT4 for 24 h followed by coculture with MCF-7 cells for 48 h. MCF-7 cells were harvested from cocultures and mRNA expression of *HK2*, *PFKFB3*, and *PDK1* was measured. Data are normalized to control MCF-7 cells cocultured with scramble transfected MΦ. Data are represented as mean ± SEM of $n \geq 3$ and p-values were calculated using Wilcoxon rank-sum test *, $p < 0.05$; **, $p < 0.01$.

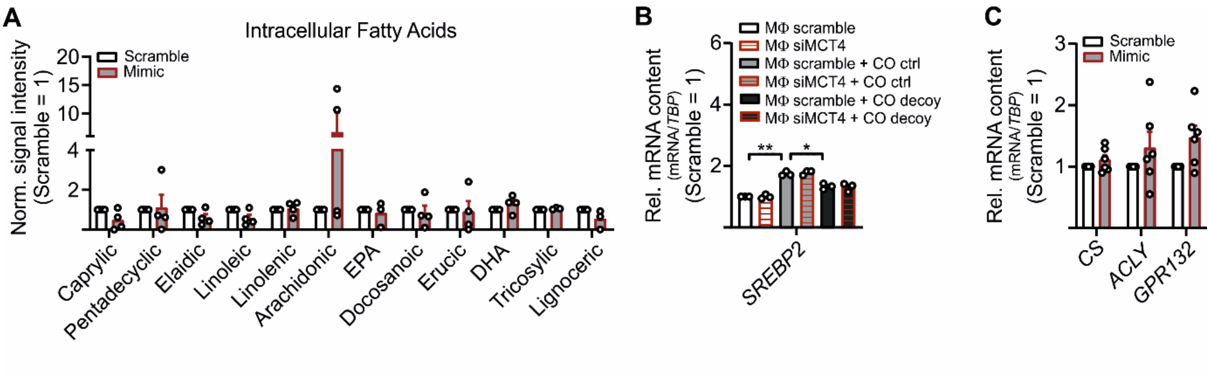

Figure S4. MiR-375-mediated LDHB downregulation induces SREBP2 activation and cholesterol biosynthesis in MΦ, related to Figure 4. (**A** and **C**) MΦ were treated with synthetic miR-375 mimic or cel-miR-39a (scramble) for 48 h. Data are normalized to scramble transfected MΦ. (**A**) Intracellular fatty acids were measured by GC/MS. (**B**) MΦ were transfected with non-specific siRNA or siRNA against *MCT4* for 24 h followed by coculture with MCF-7 cells for 48 h. MCF-7 cells were removed and *SREBP2* mRNA expression was analyzed in MΦ. Data are normalized to scramble transfected MΦ. (**C**) mRNA expression of citrate synthase (*CS*), ATP-citrate synthase (*ACLY*) and G-protein coupled receptor 132 (*GPR132*). Data are represented as mean ± SEM of $n \geq 3$ and *p*-values were calculated using Wilcoxon rank-sum test *, $p < 0.05$; **, $p < 0.01$.

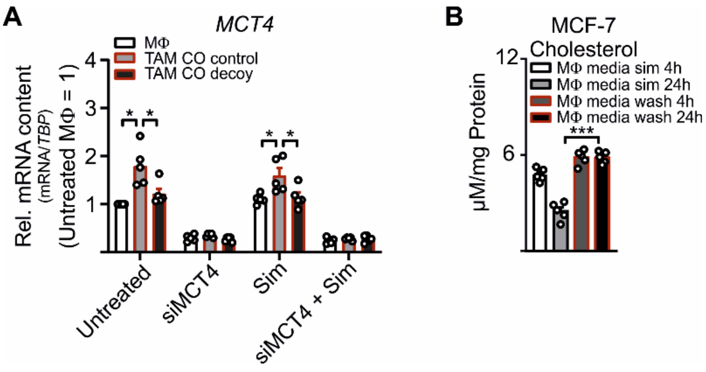

Figure S5. MΦ-derived cholesterol enhances breast tumor cell proliferation, related to Figure 5. (**A**) MΦ were transfected with non-specific siRNA or siRNA against *MCT4* for 24 h and pre-treated with 1 µM simvastatin (Sim) or DMSO for 2 h followed by coculture with MCF-7 cells for 48 h. MCF-7 cells were removed and *MCT4* mRNA expression was analyzed in MΦ. Data are normalized to scramble transfected MΦ. (**B**) Control experiment to exclude a direct effect of simvastatin remaining in the MΦ culture dishes on MCF-7 cell cholesterol biosynthesis. Primary human MΦ were treated with simvastatin containing media for 2 h and media was collected (MΦ media sim). Macrophages were washed three times with PBS and fresh macrophage media was added. After 2 h, this media was collected, too (MΦ media wash). Both, simvastatin containing media and control media were used to treat MCF-7 cells for 4 h and 24 h. An AmplexRed Cholestrol assay was performed with tumor cells. Data are represented as mean ± SEM of $n \geq 3$ and p-values were calculated using Wilcoxon rank-sum test *, $p < 0.05$; ***, $p < 0.001$.

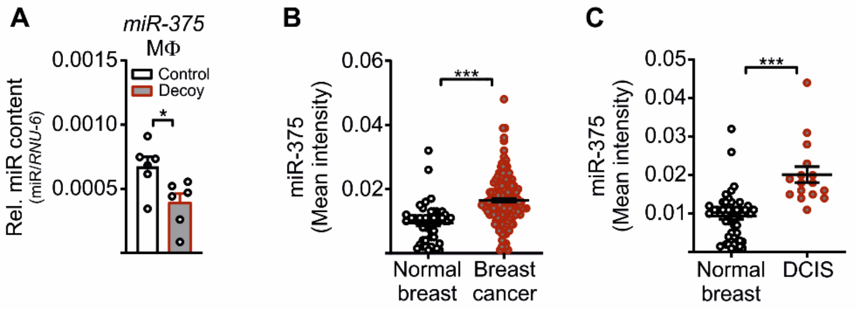

Figure S6. MiR-375 decreased LDHB *in vivo* and in human invasive breast carcinoma, related to Figure 6. (**A**) miR-375 abundance in murine MΦ sorted from MCF-7 control and decoy tumors (*n* ≥ 3). (**B**) Mean signal intensity of miR-375 in human invasive breast cancer (breast cancer) sections compared with normal breast tissue sections is shown (*n* = 156 breast tumors; n = 49 normal breasts). (**C**) Mean intensity of miR-375 in human ductal carcinoma in situ (DCIS) sections compared with normal breast tissue sections is shown (*n* = 16 DCIS tumors; *n* = 49 normal breasts). Data are represented as mean ± SEM of *n* ≥ 3. *p*-values were calculated using one-sample *t* test. *, *p* < 0.05, ***, *p* < 0.001.

Table S1. Primers used in qPCR.

Gene	sense (5' – 3')	anti-sense (5' – 3')	NCBI Gene ID
TBP	GGGCCGCCGGCTGTTTAACT	AGCCCTGAGCGTAAGGTGGCA	6908
LDHB	CCTCAGATCGTCAAGTACAGTCC	ATCACGCGGTGTTTGGGTAAT	3945
LDHA	ACGTCAGCAAGAGGGAGAAA	CGCTTCCAATAACACGGTTT	3939
PDK1	GAGAGCCACTATGGAACACCA	GGAGGTCTCAACACGAGGT	5163
HK2	TCACATGATCCCGAGATGCC	CTTGCGGAACCGCTTAGAGA	3099
PFKFB3	ATTGCGGTTTTCGATGCCAC	GCCACAACTGTAGGGTCGT	5209
MCT1	GGTGGAGGTCCTATCAGCAGT	CAGAAAGAAGCTGCAATCAAGC	6566
MCT4	CGGCTTTGTGCTTTACGCC	GCTGAAGAGGTAGACGGAGTA	9123
IL23	CAGAGAGAATCAGGCTCAAAGC	AGCAACAGCAGCATTACAGC	51561
VEGFA	CGAACGTACTTGCAGATGTGACA	GTCTTTCCTGGTGAGAGATCTGGTT	7422
ARG	GTGGAAACTTGCATGGACAAC	AATCCTGGCACATCGGGAATC	383
IL10	GACTTTAAGGGTTACCTGGGTTG	TCACATGCGCCTTGATGTCTG	3586
CLEC7A	CCGGTAAGTACCTAGCCCACA	GCTCCTGAGATGACTGTCTGT	64581
GLUT1	TCACTGTGCTCCTGGTTCTG	CCTGTGCTCCTGAGAGATCC	6513
SREBP2	AACGGTCATTCACCCAGGTC	GGCTGAAGAATAGGAGTTGCC	6721
SREBP1	GCCCCTGTAACGACCACTG	CAGCGAGTCTGCCTTGATG	6720
FASN	AAGGACCTGTCTAGGTTTGATGC	TGGCTTCATAGGTGACTTCCA	2194
ACACA	GCTTGCCTGACTTTTGATCCG	ACGTTATCCCCAAACCCAGG	31
ACACB	CAAGCCGATCACCAAGAGTAAA	CCCTGAGTTATCAGAGGCTGG	32
LDLR	TCTGCAACATGGCTAGAGACT	TCCAAGCATTCGTTGGTCCC	3949
HMGCS	CTCTTGGGATGGACGGTATGC	GCTCCAACTCCACCTGTAGG	3157
HMGCR	TTCGGTGGCCTCTAGTGAGA	TGTCACTGCTCAAAACATCCTCT	3156
MVK	GGGGATTGCGTCAACAGGT	GGGTTCCCGTGAATCATTCTC	4598
CS	TGCTTCCTCCACGAATTTGAAA	CCACCATACATCATGTCCACAG	1431
ACLY	GATTTTGCGGGGTTCGTCG	TTGCCCGTCTGCTCTGAAAT	47
GPR132	TGTTCCAGACGGAAGACAAGG	GCGTAGTAGTACCCGGCAA	29933
pre-miR-375	CCGCGACGAGCCCCT	CCGAACGAACAAAACGCTCA	494324
Ldhb	CATTGCGTCCGTTGCAGATC	GGAGGAACAAGCTCCCGTG	16832

Ldha	ACATTGTCAAGTACAGTCCACAC	TTCCAATTACTCGGTTTTTGGGA	16828
Rps27a	GACCCTTACGGGGAAAACCAT	AGACAAAGTCCGGCCATCTTC	78294
Rnu6	CTTCGGCAGCACATATACTAAAAT		19862
